# Rediscovering the unusual, solitary bryozoan *Monobryozoon ambulans* Remane, 1936: first molecular and new morphological data clarify its phylogenetic position

**DOI:** 10.1186/s12983-024-00527-1

**Published:** 2024-03-05

**Authors:** Thomas Schwaha, Sebastian H. Decker, Christian Baranyi, Ahmed J. Saadi

**Affiliations:** Department of Evolutionary Biology, University of Vienna, Schlachthausgasse 43, 1030 Vienna, Austria

**Keywords:** Meiofauna, Heligoland, Solitary, Phylogenetic relationship, Solitary bryozoa, Mitogenome

## Abstract

**Background:**

One of the most peculiar groups of the mostly colonial phylum Bryozoa is the taxon *Monobryozoon*, whose name already implies non-colonial members of the phylum. Its peculiarity and highly unusual lifestyle as a meiobenthic clade living on sand grains has fascinated many biologists. In particular its systematic relationship to other bryozoans remains a mystery. Despite numerous searches for *M. ambulans* in its type locality Helgoland, a locality with a long-lasting marine station and tradition of numerous courses and workshops, it has never been reencountered until today. Here we report the first observations of this almost mythical species, *Monobryozoon ambulans*.

**Results:**

For the first time since 1938, we present new modern, morphological analyses of this species as well as the first ever molecular data. Our detailed morphological analysis confirms most previous descriptions, but also ascertains the presence of special ambulatory polymorphic zooids. We consider these as bud anlagen that ultimately consecutively separate from the animal rendering it pseudo-colonial. The remaining morphological data show strong ties to alcyonidioidean ctenostome bryozoans. Our morphological data is in accordance with the phylogenomic analysis, which clusters it with species of *Alcyonidium* as a sister group to multiporate ctenostomes. Divergence time estimation and ancestral state reconstruction recover the solitary state of *M. ambulans* as a derived character that probably evolved in the Late Cretaceous. In this study, we also provide the entire mitogenome of *M. ambulans*, which—despite the momentary lack of comparable data—provides important data of a unique and rare species for comparative aspects in the future.

**Conclusions:**

We were able to provide first sequence data and modern morphological data for the unique bryozoan, *M. ambulans*, which are both supporting an alcyonidioidean relationship within ctenostome bryozoans.

**Supplementary Information:**

The online version contains supplementary material available at 10.1186/s12983-024-00527-1.

## Background

Bryozoa is a phylum of lophotrochozoans most likely closely related to other lophophorate phyla, Brachiopoda and Phoronida (see Bleidorn [1] for a recent review on lophotrochozoan phylogeny). In contrast to the other two lophophorates, bryozoans are generally colonial, having numerous interconnected individuals called zooids that form the colony [2]. Each zooid has an outer protective body wall, traditionally termed cystid, and soft-tissues, such as the lophophore and U-shaped digestive tract, which are termed polypide. In addition to coloniality, the retractability of the polypide into the cystid is the second main apomorphic character of bryozoans [3].

Bryozoa is a large phylum with over 6000 Recent described species [4], most of them belonging to clades Cheilostomata or Cyclostomata, which form mineralized, calcified skeletons. Ctenostome bryozoans are a small group of animals lacking calcified skeletons. Within this group there are two families that are considered as solitary [5, 6]. Solitary is defined here as always consisting of a single feeding zooid with initially polymorphic zooids that most likely separate from the mother zooid. Aethozoidae comprises single feeding zooids with a limited number of thin appendages, some of them as part of the zooid, cystid appendages, some as polymorphs, so-called kenozooids in bryozoans. The latter are probably asexual budding stages—a feature very common in colonies and their growth.

The other family of solitary bryozoans is Monobryozoidae, a cryptic clade of bryozoans that live on soft surfaces at shallow depths [7]. The type species of the family is *Monobryozoon ambulans*, a species originally described in the beginning of the twentieth century from Helgoland, in the North Sea [8]. Its single feeding zooid has several proximal appendages, termed ambulatory processes owing to their ability to move via muscular contractions that enable animals to crawl, move or reorientate themselves on the sandy substrates they live upon.

Ever since its initial finding and description by Adolf Remane, only a second sighting was reported from the type locality [9], and a single observation found it on the East coast of Great Britain [10]. A second species of *Monobryozoon* was discovered in 1972 from the East coast of Northern America [7, 11], whereas the report of a third species is highly dubious [12].

From a morphological perspective both solitary families are considered unique, but also still little investigated [7]. From the currently available data it is not possible to draw any proper conclusions as to the systematic relationship of both of the families. Recent morphological data is available for one aethozoid [6], but monobryozoids remain more or less unstudied. Sequence data establishing the phylogenetic position of these aberrant forms is also entirely missing for both.

Despite numerous searches for *Monobryozoon ambulans* at its type locality, the species had not been reencountered since its second finding in 1938. This also included a dedicated two week search by the first author, which was funded by the EU programme Assemble Plus. However, during a subsequent marine biological course of the University of Vienna in Helgoland, we encountered numerous specimens of *M. ambulans*. This exciting discovery allowed us to preserve this almost unknown species for various approaches. In this study, we report the first finding of *M. ambulans* from its type locality since 1938, and also reveal new morphological data for this species. We provide the entire mitogenome and also a transcriptome for phylogenetic reconstructions. This multi-disciplinary approach allowed us to unravel the enigmatic position of monobryozoids in the phylogenetic system for the first time. Lastly, comparative morphological analysis between monobryozoids and aethozoids was used to evaluate possible independent origins of a solitary lifestyle among bryozoans.

## Materials and methods

### Sample collection and preservation

Samples were collected by bottom sampling at 54°15′04.7″N, 7°56′48.8E at ~ 40 m depth in April 2022. Sand samples were filtered as described by Gray [10]. Collected monobryozoans were partially documented on site with a Leica M165C (Leica Microsystems, Wetzlar Gemany) equipped with a Leica ICE90 camera. Most samples were fixed either in 2% glutaraldehyde in 0.1 M phosphate buffer until further procedure in Vienna, or in 4% paraformaldehyde in 0.05 M phosphate buffer for 1 h followed by several rinses in the buffer prior to staining in Vienna. For molecular approaches, a few specimens were fixed in absolute ethanol or RNALater.

### Morphological analyses

#### Histology and 3D reconstruction

Glutaraldehyde-fixed specimens were processed for sectioning as described by e.g. Schwaha [13] and afterwards sectioned at 0.5–1 μm section thickness on a Leica UC6 ultramicrotome. Series of sections were stained with toluidine blue and sealed in Agar Low Viscosity resin. Sections were visualized and photographed with a Nikon NiU (Nikon, Tokyo, Japan) microscope equipped with a Nikon Ri2 camera. Image stacks were modified and converted to grayscale with FIJI [14] prior to import into the software Amira 2021.1 (ThermoFisher). There, sections were aligned and segmentations of various organs were conducted (see [15] for details). Reconstructions were visualized as snapshots in the software.

#### Phalloidin staining and scanning

Paraformaldehyed-fixed specimens were first incubated into 2% Triton-X, 2% dimethylsufloxide in 0.1 M phosphate buffer for permeabilization for almost 24 h for permeabilization. Afterwards, a 1:40 diluted concentration of the Alexa Fluor™ 488 Phalloidin (Thermo Fisher Scientific, Waltham MA, USA) was added to the permeabilization solution was used for staining f-actin. Stained samples were rinsed 3–4 times in phosphate buffer for 20 min each prior to mounting of the samples in FluoromountG (Sigma) and subsequent analysis using a Leica SP5 II confocal microscope. Confocal image stacks were analysed with FIJI or Amira.

### Molecular analysis

#### RNA extraction, library construction and sequencing

RNA was extracted from two species (*Monobryozoon ambulans* and *Paludicella articulata* as additional ctenostome to extend available transcriptomes. *P. articulata* was collected in Laxenburg, Lower Austria) preserved in RNAlater. For *M. ambulans*, RNA was extracted using the RNAqueous™-Micro Total RNA Isolation Kit (Thermo Fisher Scientific, Waltham MA, USA) following the manufacturer’s instructions. For *P. articulata*, RNA was extracted using the RNeasy® Plus Mini kit (QIAGEN, Hilden, Germany) following the manufacturer’s instructions. Library preparation and sequencing were performed by the Next Generation Sequencing Facility at Vienna BioCenter core Facilities (VBCF), member of the Vienna BioCenter (VBC). For *M. ambulans*, dual-indexed sequencing libraries were prepared using the SMART-Seq v3-Low Input RNA-seq kit. For *P. articulata*, dual-indexed sequencing libraries were constructed using the NEBNext® UltraTM II Directional RNA Library Prep Kit (#E7760, New England Biolabs, Frankfurt am Main, Germany) according to the manufacturer’s instructions. The samples were then multiplexed and sequenced on an Illumina NovaSeq 6000 using an S4 flowcell with 2 X 150 bp paired-end reads with the S2 protocol at the VBCF or Psomagen, Inc. (Cambridge, MA, USA).

#### Transcriptome assembly and functional annotation

Raw Illumina reads were quality-checked before and after trimming using FastQC v0.11.8 (www.bioinformatics.babraham.ac.uk/projects/fastqc; last accessed April 08, 2022). Adapters and low-quality reads were removed from the raw Illumina reads using Trimmomatic v0.39 [16] with default parameters, and then clean reads were de novo assembled in Trinity v2.8.4 [17], under default settings, with the exception of a minimum transcript length of 200 nucleotides. The assembled transcriptomes were screened for possible contamination using a custom BLAST search against a database composed of nine genomes of protists and diatomic algae as described in Khalturin et al. [18]. Transdecoder v5.02 (https://github.com/TransDecoder/TransDecoder/; last accessed April 08, 2022) with the -single_best_only option was used to predict coding sequences and translate the longest open reading frames (ORFs) into peptide sequences. Only ORFs that were at least 100 amino acids long were retained. To reduce redundancy in the predicted peptides, CD-HIT v4.8.1 [19] was applied using a threshold of 95% global similarity. Finally, the completeness of the transcriptomes was assessed using BUSCO v5.2.2 [20] with default settings against the conserved single-copy metazoan genes database (n = 954).

#### Orthologue assignment, alignment and matrix construction

Putatively orthologous groups (OGs) shared among taxa were inferred using OrthoFinder v2.5.2 [21] with an inflation parameter of 2.1. Orthogroups produced by the OrthoFinder “Orthogroup_Sequences” directory were processed using a modified version of the pipeline employed by Kocot et al. [22] as described in Saadi et al. [23]. First, sequences that were identical to longer sequences where they overlapped were removed from each orthogroup (keeping the longest non-redundant sequence). Only orthogroups present in at least 75% of the sampled species were retained and aligned using MAFFT 7.310 [24] with the following options: -auto, -localpair, and -maxiterate 1000. Putatively mistranslated regions were removed using HmmCleaner [25] with the -specificity option and alignments were trimmed with BMGE v. 1.12.2 [26] to remove ambiguously aligned and ‘noisy’ regions. Sequences that did not overlap with all other sequences by at least 20 amino acids sequences (AAs) were deleted using AlignmentCompare (https://github.com/kmkocot/basal_metazoan_phylogenomics_scripts_01-2015/; last accessed April 08, 2022). Only genes sampled for a minimum of 20 taxa after these steps were retained. Maximum likelihood trees were constructed for these genes using FastTree 2 [27] with the -slow and -gamma settings. Strictly orthologous sequences among taxa were identified using PhyloPyPruner 0.9.5 (https://pypi.org/project/phylopypruner/; last accessed April 08, 2022) with the following settings: -min-support 0.9 -mask pdist -trim-lb 3 -trim-divergent 0.75 -min-pdist 0.01 -prune LS. Using this pipeline, we identified 2,014 OGs which are present in at least 75% of the sampled species (i.e. 20 taxa) which were then used to make the “complete dataset”. To assess the effects of relative composition frequency variability (RCFV) on phylogenetic analyses, we calculated the normalized RCFV (nRCFV) using nRCFV_Reader [28] for each OG. The nRCFV accounts for biases in RCFV caused by sequence length, the number of taxa, and the number of character states within dataset [28]. Generally, larger values of nRCFV are “worse” (more likely to cause systematic error) than smaller values. We selected the best 1,500 OGs based on nRCFV values and concatenated them with FASconCAT [29] to make the “subsampled dataset” which was analysed separately from the complete dataset.

#### Phylogenetic analyses and assessment of model fit

Maximum likelihood (ML) phylogenetic inference was performed on amino acid sequences of the two matrices (the complete and the subsampled data matrices) using IQ-TREE2 v2.1.4 [30]. One ML analysis for each dataset was undertaken with option -m MFP + MERGE using ModelFinder [31] in IQ-TREE2 to select the best partition scheme and best model for each partition. Topological support was assessed with 1,000 ultrafast bootstraps. A Second ML analysis was performed on both datasets using the posterior mean site frequency (PMSF) model (LG + C60 + G + F) [32] in IQ-TREE2; the previously generated ML tree using the best partition scheme was used as a guide tree for PMSF analysis. Topological support was assessed with 1,000 replicates of ultrafast bootstraps.

Bayesian inference (BI) was performed with PhyloBayes MPI [33] on amino acid sequences of the two data matrices using the site-heterogeneous CAT-F81 + G4 and CAT-GTR + G4 models. BI analyses were run with two parallel chains. For the complete data matrix, the chains were run for 21,000 cycles each, with the first 2,500 trees discarded as burn-in. For the subsampled data matrix, the chains were run for 8,000 cycles each, with the first 1,000 trees discarded as burn-in. For both matrices, a 50% majority rule consensus tree was computed from the remaining trees from each chain. Convergence of the PhyloBayes chains was assessed by inspection of the tracefile outputs in Tracer [34] and based on the tracecomp and bpcomp commands in PhyloBayes. In the complete data matrix, both analyses (CAT-F81 + G4 and CAT-GTR + G4) showed an acceptable degree of convergence (effective sample size > 100, relative differences < 0.3), though the maxdiff value was > 0.3 “ = 1 indicating that the analyses had not converged according to this strict measure”. While in the subsampled data matrix, the two analyses showed a good degree of convergence (the effective sample size > 100, relative differences < 0.3 and maxdiff value = 0.006 for the CAT-F81 + G4 and maxdiff value = 0 for the CAT-GTR + G4).

We assessed the absolute fit of different substitution models to the data for both matrices using posterior predictive analyses (PPA) implemented in PhyloBayes MPI 1.9 [33, 35]. Five statistical measures of PPA were tested, three of them (PPA-DIV, PPA-CONV, and PPA-VAR) assessed modelling of site-specific heterogeneity [33, 35] while the remaining two (PPA-MAX and PPA-MEAN) assessed modelling of lineage specific heterogeneity [36]. The fit of each model was evaluated by calculating the absolute *Z*-score for each statistic using observed and simulated data. The *Z*-score represents the number of standard deviations by which the simulated data deviates from the observed mean. If a Z-score is less than two, it indicates that the model fit the data adequately, while a *Z*-score larger than five indicates that the model cannot adequately fit the data [25, 37]. PPA were performed on the two matrices using PhyloBayes-MPI to test whether the site-homogeneous models (GTR + G4, LG + G4) and the site-heterogenous models (CAT-GTR + G4, CAT-F81 + G4) adequately describe site-specific amino acid heterogeneity. The BI analyses were performed with a single chain run for at least 5,000 iterations to generate enough replicates to be representative of each chain. PPA results were then obtained for each run using the “–allppred” flag in readpb_mpi with a burn-in of 1,000 iterations and sampling every 10 iterations.

#### Divergence time estimation and ancestral state reconstructions (ASR)

To estimate divergence times, we used MCMCTree and codeml, both part of the PAML software package, v. 4.9 [38] with the independent rates model (clock = 2). The complete dataset was utilized for this analysis and the ML tree obtained from the best partition scheme was used as the input tree. The input tree was calibrated using age estimates of five fossils that were also used by Saadi et al. [23]. For the MCMCtree analysis, three different calibration strategies were used. They were: (1) the truncated-Cauchy distribution ‘L’; (2) the skew normal ‘SN’; and (3) the uniform distribution ‘B’. The R package MCMCtreeR [39] was used to construct calibration densities for these three strategies (for details of fossil calibration points and divergence time analysis see Additional file 1, methods section two).

Ancestral State Reconstructions (ASR) for bryozoan lifestyles was performed in Mesquite 3.70 [40] using the “Trace Character History” and “Likelihood Ancestral States” options with the Mk1 model. The ML tree inferred based on the best partition scheme from the complete data matrix was used as input tree for the ASR analysis. A morphological matrix for all analysed bryozoan species based on lifestyles was compiled including two states (colonial and solitary).

#### DNA extraction, mitochondrial genome sequencing and assembly and annotation

DNA of *M. ambulans* was extracted from ethanol preserved specimen using QIAmp DNA Micro Kit (QIAGEN, Hilden, Germany) following the manufacturer’s instructions. Library preparation and sequencing were performed by the Next Generation Sequencing Facility at VBCF, member of the VBC. Briefly, genomic DNA libraries were constructed using NEBNext® Ultra™ II FS DNA Library Prep Kit for Illumina, with Imputs ≥ 100 ng (# E7805) and **NEBNext Multiplex Oligos for Illumina (**Dual Index Primers, NEB #E7600). Libraries were multiplexed and sequenced on an Illumina NextSeq 550 platform using the 300 Cycle Mid Output mode.

Raw Illumina reads were quality-checked before and after trimming using FastQC v0.11.8 (www.bioinformatics.babraham.ac.uk/projects/fastqc; last accessed April 08, 2022). Reads were trimmed of adapters and low-quality sequences using Trim Galore v0.6.5 (https://github.com/FelixKrueger/TrimGalore; last accessed April 08, 2022) with default setting. The retained filtered reads were de novo assembled using SPAdes v3.15.3 [41] with k-mers of 21, 33, 55, 77, 99 and 127. The mitogenome was identified using BLASTN [42] and annotated with MITOS2 web server [43] using a metazoan reference (RefSeq 89) and the invertebrate genetic code. Manual curation of the mitogenome was undertaken using the mitogenomes of *Bugula neritina* (AY690838) [44] and *Flustrellidra hispida* (NC_008192) available at NCBI as references. A circular map of the *M. ambulans* mitogenome was generated with OrganellarGenome-DRAW (OGDRAW) online server [45].

## Results

### Morphological data

**Fig. 1:**
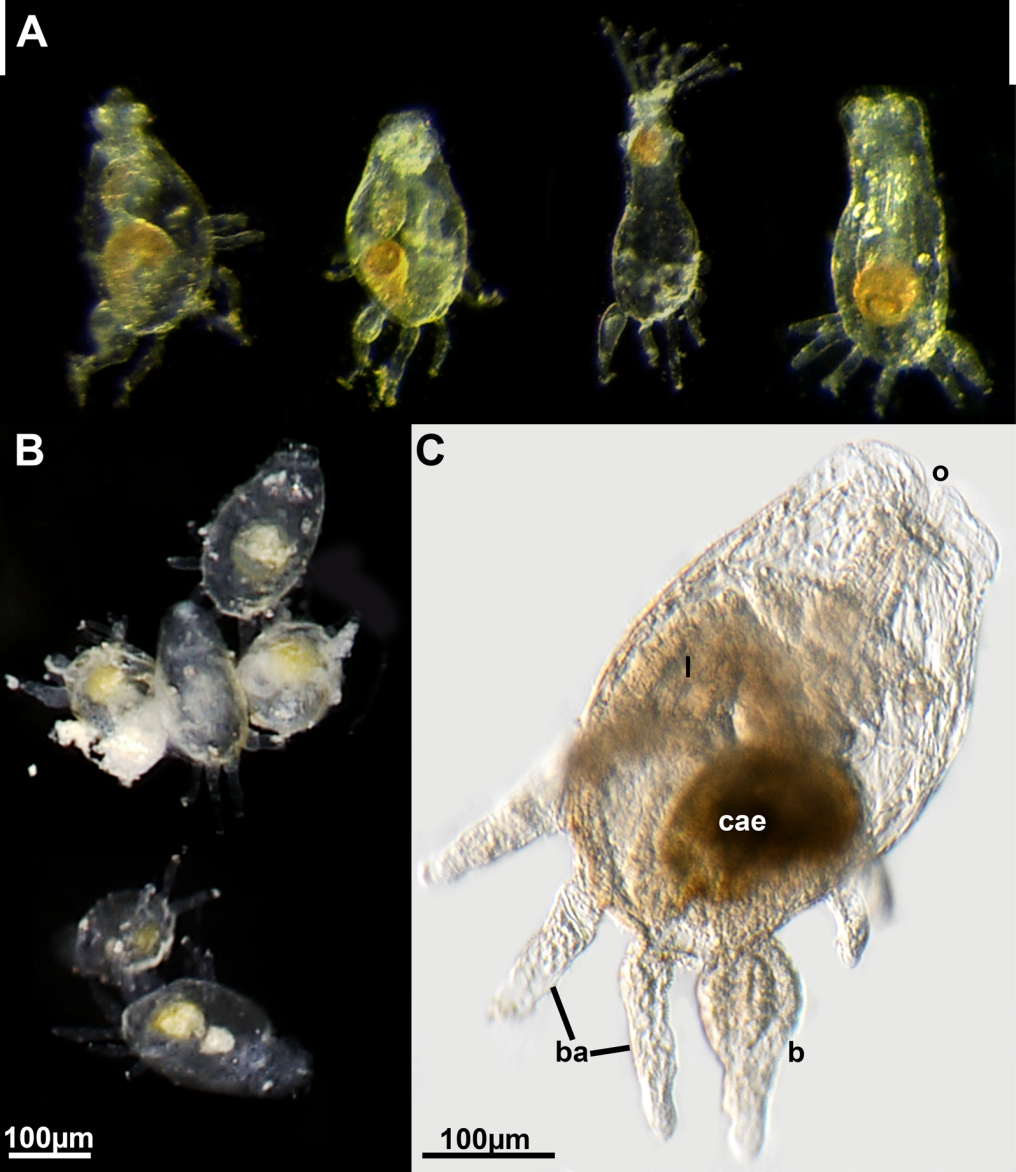
*Monobryozoon ambulans*. **A** Unfixed specimens. Zooidal width equals approximately 100 µm. The specimen with extended lophophore is dead. **B** A series of fixed monobryozooids. **C** Close-up of a mounted specimen. b—bud, ba—basal appendage, cae—caecum, l—lophophore, o—orifice

**Fig. 2:**
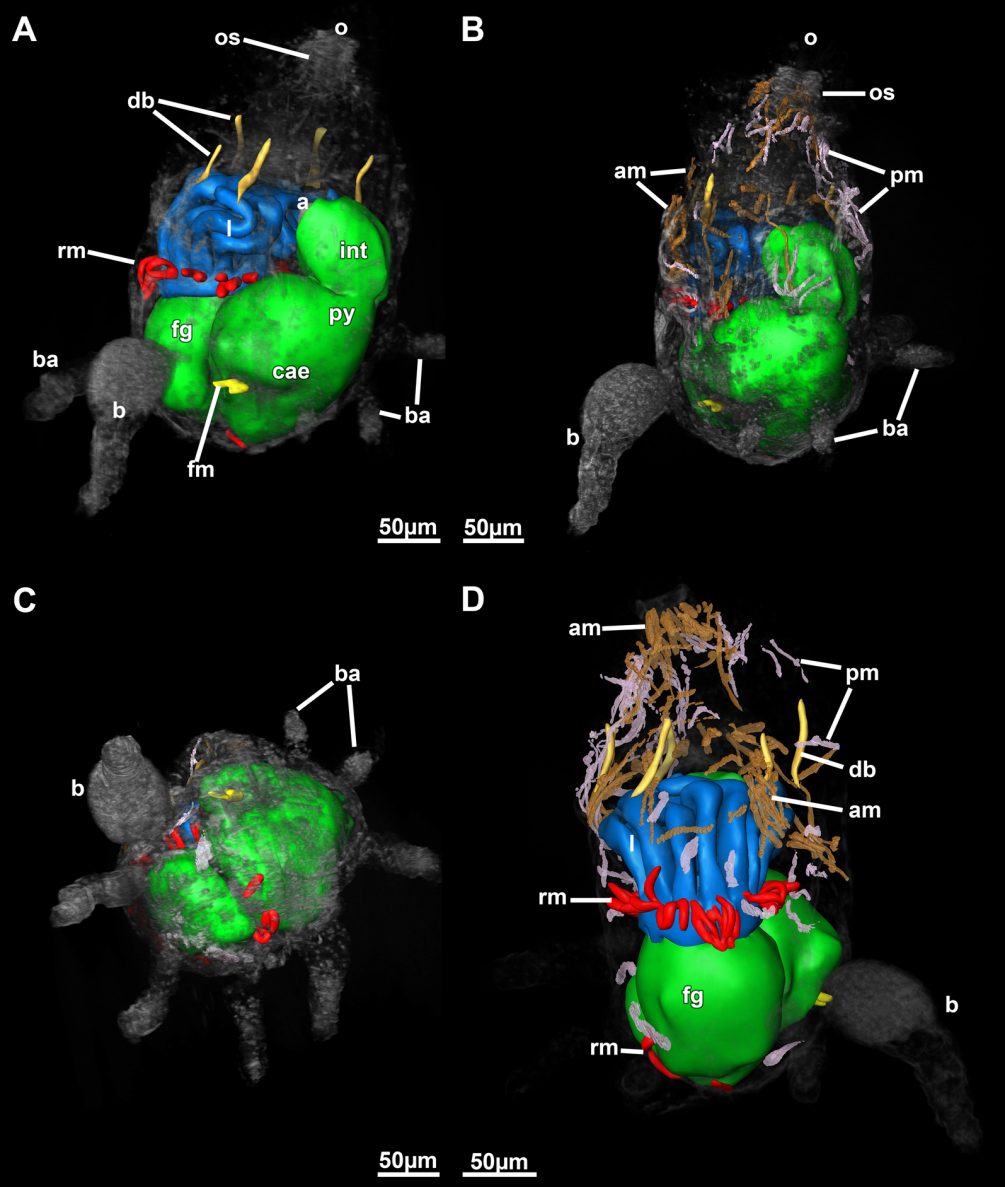
3D-reconstruction based on histological serial sections of *Monobryozoon ambulans*. **A** Lateral view showing the main parts of the lophophore and digestive tract. In addition, duplicature bands, funicular and retractor muscles are displayed. **B** Lateral view similar to A but showing parietal and apertural muscles. **C** Basal view showing the different appendages. **D** Lateral view with the body wall more transparently showing the distal muscle systems more clearly. a—anus, am—apertural muscles, b—bud, ba—basal appendage, cae—caecum, db—duplicature band, fg—foregut, fm—funicular muscle, int—intestine, l—lophophore, o—orifice, os—orificial sphincter, pm—parietal muscles, py—pylorus, rm—retractor muscles

**Fig. 3:**
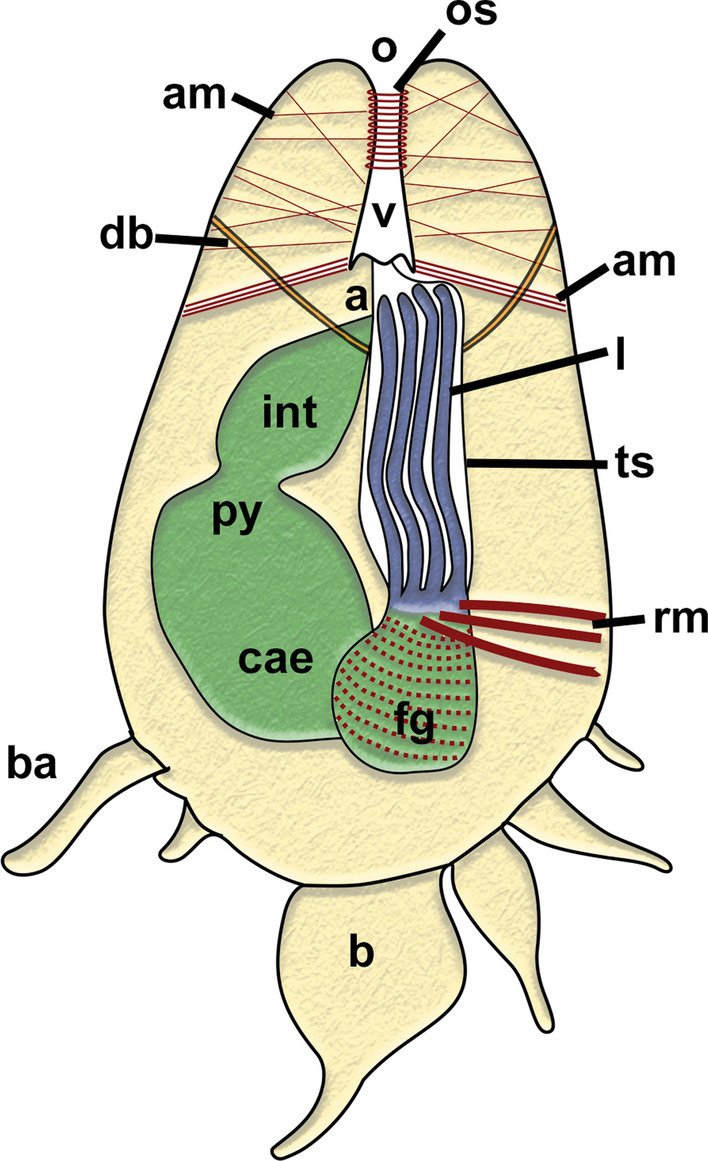
Schematic drawing of a retracted zooid of *Monobryozoon ambulans*. a—anus, am—apertural muscles, b—bud, ba—basal appendage, cae—caecum, db—duplicature band, fg—foregut, int—intestine, l—lophophore, o—orifice, os—orificial sphincter, py—pylorus, rm—retractor muscles, ts—tentacle sheath, v—vestibulum

**Fig. 4:**
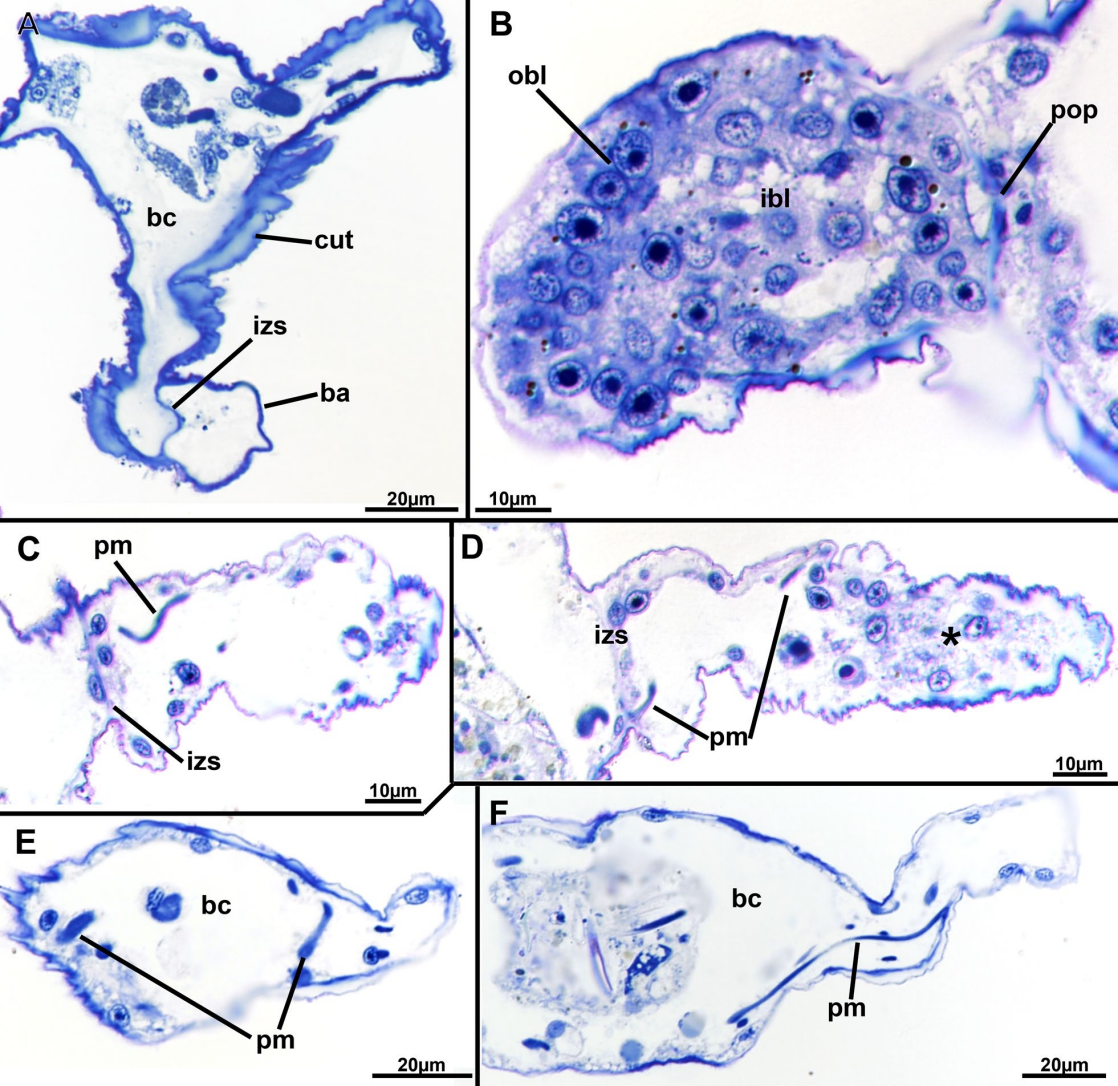
Histological details of the basal areas and appendages of *Monobryozoon ambulans*. **A** Cross-section of the proximal area showing the main body cavity, thick cuticle, and an interzooidal septum between the basal appendage and the main body cavity. **B** Longitudinal section of a basally enlarged appendage showing the proliferating, budding cells. **C**, **D** Two different sections of basal appendages showing the parietal muscles enclosed within the appendage, past the interzooidal septum. Asterisk shows clusters of cells in the more distal part of the appendage. **E**, **F** Two different sections showing the traverse of parietal muscles within the main body cavity of the zooid. ba—basal appendage, bc—body cavity, cut—cuticle, ibl—inner budding layer, izs—interzooidal septum, obl—outer budding layer, pop—pore plate, pm—parietal muscles

**Fig. 5:**
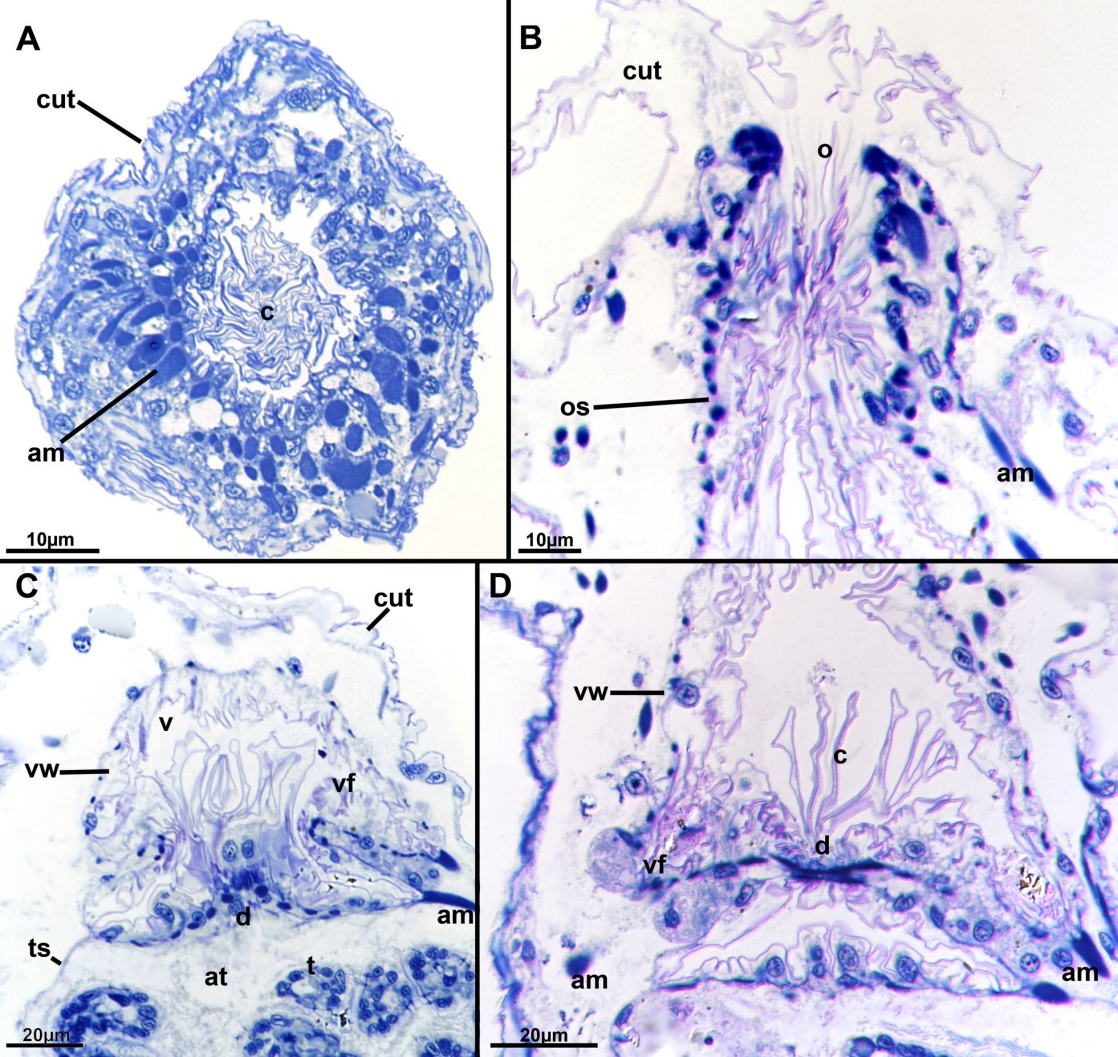
Histological details of the apertural area of *Monobryozoon ambulans*. **A** Cross-section of the distal apertural area showing a massive collar within the vestibulum and dense apertural muscles. **B** Longitudinal muscles showing the dense orificial sphincter and the distal, thick cuticle. **C** Diaphragmatic sphincter showing the distinct vestibular folds. **D** Detail of the diaphragm showing lateral expansions of the vestibular epithelium and corresponding muscles. Note the more prominent cells lining the vestibular fold. am—apertural muscles, at—atrium, c—collar, cut—cuticle, d—diaphragm, o—orifice, os—orifical sphincter, t—tenacle, ts—tenacle sheath, v—vestibulum, vf—vestibular fold, vw—vestibular wall

*Monobryozoon ambulans* forms sac-shaped zooids with several basal thin appendages. There are usually eight to nine appendages (Figs. 1A, C, 2C, 3) with one showing a proximal expansion indicating the formation of an early, asexual bud that in all investigated specimens shows a two-layered vesicle stage (Fig. 4B). A cuticular septation is present at the attachment site of each appendage, which separates its cavity from the remaining zooid (Fig. 4A, C, D). Specific pore cell complexes were only detected on the bud appendage (Fig. 4B), but given its small size of 1–2 µm on sections, it could easily be overlooked or require higher resolutions such as electron microscopy to distinguish. Distinct glandular patches were not observed in any part of the appendage and the epidermis is equally thin with a thin cuticle. On the main zooid, the cuticle is rather thick and shows surface increases (Fig. 5A). In some appendages an increased number of cells was encountered (Fig. 4D), but this does not promote the idea of any specific adhesive system present in such appendages.

**Fig. 6:**
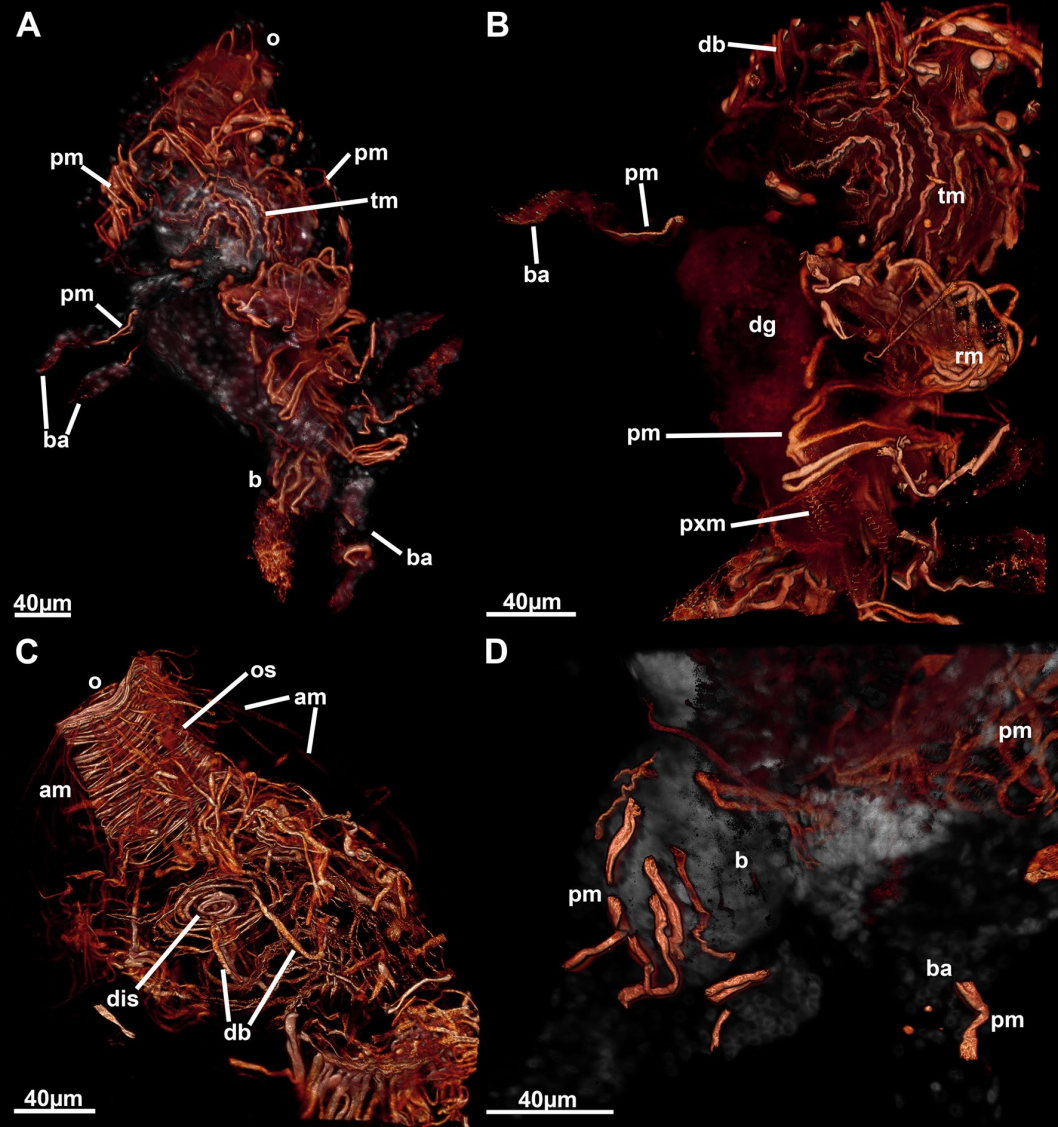
Myoanatomical detail of *Monobryozoon ambulans* based on phalloidin staining and confocal microscopy. **A** Lateral view showing the main concentrations of the main muscle systems. **B** Detail of the lophophoral base and foregut. **C** Detail of the apertural area showing the orificial sphincter. **D** Detail of the basal appendages showing individual muscles not connected to the main body cavity. am—apertural muscles, b—bud, ba—basal appendage, db—duplicature band, dg—digestive tract, dis—diaphragmatic sphincter, o—orifice, os—orificial sphincter, pm—parietal muscles, pxm—pharynx musculature, rm—retractor muscles, tm—tentacle muscles

Appendages of *M. ambulans* show distinct muscular bands traversing the cavity as short bands in the proximal area allowing a certain mobility to crawl on sand particles. These muscle fibres are not continuous with the main body cavity of the autozooid, but remain entirely within the appendage (Figs. 4C, D; 6A, D). Compared to the main zooidal cavity, the muscles encountered in the appendages are parietal muscles (Figs. 4E, F; 6A, D). In the appendages, these extend from two closely-located areas of the appendage cavity, spanning only a short distance and never traversing the cavity directly (Figs. 4E, F, 6D). Within the main zooidal cavity harbouring the polypide, the parietal muscles have a similar extension and are mere thin muscle fibres located in multiple parts of the body wall.

The remaining muscular systems shows particular concentrations in the apertural area, specifically the orifice where the tentacles of live zooids emerge. In retracted zooids a particularly strong orifical sphincter is present in this protective area (Figs. 5B, 6C). Concerning the remaining musculature, a series of parietodiaphragmatic muscles is also present that insert at the diaphragm—the transition of the vestibular wall to the tentacle sheath. Five duplicature bands—peritoneal muscular bands connecting the tentacle sheath with the body wall—are present in *M. ambulans* (Figs. 2, 6C). Tentacle musculature is discernible (Fig. 6B), but musculature of the digestive tract is minimal except the foregut.

The lophophore of *M. ambulans* carries 12 tentacles. In the retracted condition it is encased within the tentacle sheath in the basal half of the zooid, whereas the distal part consists entirely of the apertural area (Fig. 2). At the lophophoral base, the mouth opening enters the short foregut that more or less immediately enters the bulbous caecum, which itself continues with the intestine with a vestibular anus into the tentacle sheath close to the vestibular wall (Fig. 2). In live, but also fixed animals, the caecum is most prominent showing a yellow to brown coloration (Fig. 1).

The proximal area of the vestibular wall shows distinct vestibular folds, which also reflect the attachment sites of the apertural muscles (Fig. 5C, D). The specific attachment of these contracted muscles forms the described grooves in *M. ambulans.* We detected the inclusion of mineralized particles in this groove, and some cells lining that groove are very hypertrophied and prominent, which indicates a specific function or role in this area (Fig. 5D). At the diaphragm (transition from the tentacle sheath and vestibular wall) a prominent, folded collar extends as cuticular protrusion into the vestibulum. It obstructs almost the entire cavity and reaches almost the distal orifice (Fig. 5).

### Molecular data

#### Data matrices

We sequenced and assembled the transcriptomes of *M. ambulans* and *P. articulata* and combined them with publicly available transcriptomes of 21 bryozoan species. Two phoronids and two brachiopods were included as outgroups. Details of the specimens, GenBank Sequence Read Archive (SRA) accession numbers and sources of publicly available sequences are given in Additional file 1: Table S1. We also provided functional annotations of the *M. ambulans* transcriptome (details of the methodologies, assembly statistics and functional annotations of *M. ambulans* are given in Additional file 1: Table S6 and Table S7). For phylogenetic analyses, we have assembled two different matrices (the complete and the subsampled data matrices). The complete data matrix included 2,014 OGs totaling 422,961 amino acid positions with 23% missing data and nRCFV value of 0.0109 while the subsampled matrix resulted in a matrix of 1500 OGs totalling 310,190 amino acids with 19.13% missing data and nRCFV value of 0.0077.

### Phylogenetic analyses and posterior predictive analysis

**Fig. 7:**
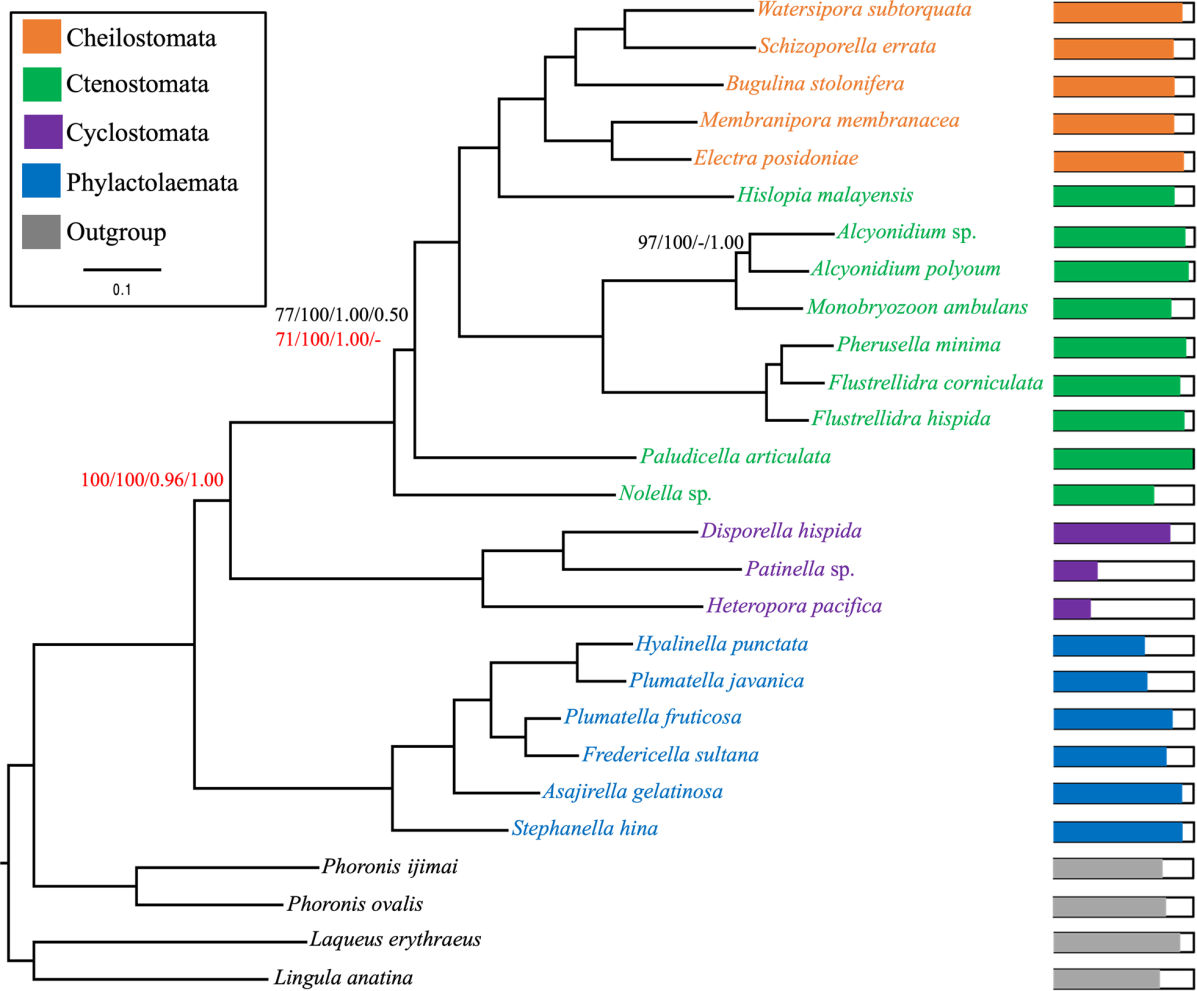
Maximum likelihood analysis of the complete data matrix, including 422,961 AAs from 2,014 OGs using partitioned analysis. Values on some nodes represent branch support (ML ultrafast bootstrap branch support of the partitioned analysis, ML ultrafast bootstrap support of the unpartitioned analysis with PMSF model, Bayesian posterior probabilities of the CAT-F81 + G4 analysis and Bayesian posterior probabilities of the of CAT-GTR + G4 analysis, respectively) values in black for the main data matrix and in red for the subsampled data matrix. Dashes indicate nodes not present. Bootstrap support and Bayesian posterior probabilities are only shown for nodes that are not maximally supported by all analyses. Coloured bars show the proportion of genes sampled for each taxon. The scale bar represents 1 substitutional change per 100 AAs

Except where noted, highly congruent tree topologies were inferred from all ML and BI analyses based on both data matrices with most nodes receiving maximal support (BI posterior probability, PP = 1.00 and ML bootstrap support, BS = 100). The ML analysis of the complete data matrix based on the best partition scheme is shown in Fig. 7. The ML analysis based on PMSF model, and BI analyses based on the CAT + F81 + G4 and CAT-GTR + G4 models, all based on the complete dataset, are shown in Additional file 1: Figures S1–S3. Phylogenetic trees of the subsampled data matrix are given in the Additional file 1 as follows: ML analysis based on the best partition scheme (Fig. S4), ML analysis based on PMSF model (Fig. S5), BI analysis under the CAT + F81 + G4 model (Fig. S6) and BI analysis under the CAT-GTR + G4model (Fig. S7). Our phylogenies strongly support a sister group relationship between Phylactolaemata and Myolaemata. Within the latter clade, Ctenostomata is shown to be a paraphyletic group and Gymnolaemata is recovered as a sister group to Stenolaemata. In all the phylogenetic analyses, we recovered *M. ambulans* as the sister taxon to a clade comprising two *Alcyonidium* species with maximal support except in the BI analysis of the complete dataset using the CAT + F81 + G4 model, in which *M. ambulans* was recovered as the sister taxon to *Alcyonidium polyoum,* though without significant support (PP = 0.51). Furthermore, the BI analysis did not show a good degree of convergence based on maxdif score (= 1).

The PPA results revealed that the site-heterogenous model CAT-F81 + G4 describes among-site amino acids preferences (based on PPA-DIV and PPA-VAR) and among-lineage compositional heterogeneity (PPA-MAX and PPA-MEAN) better than any other model for both matrices (Table 1). The CAT-GTR + G4 model was the second-best modelling strategy. *Z* scores PPA-CON for the complete and the subsampled data matrix were 9.16 and 7.22, respectively. Additionally, our PPA results of both data matrices under all models showed statistically significant compositional non-stationarity in *M. ambulans* (Additional file 1; Table S2). However, we found that the subsampled dataset showed improved model fit under PPA statistics, particularly for PPA-MEAN and PPA-MAX.

**Table 1:** Comparing model adequacy for the complete and subsampled data matrices using PPA

PPA Model	PPA-DIV	PPA-CONV	PPA-VAR	PPA-MEAN	PPA-MAX
Complete dataset					
GTR	113.25	39.52	41.29	560.711	87.12
LG	145.23	48.09	51.58	520.407	82.59
CAT_GTR	**1.95**	9.16	9.343	175.234	20.23
CAT_F81	** − 6.24**	22.47	** − 1009.3**	133.18	7.015
Subsampled dataset					
GTR	94.04	37.13	37.23	298.55	59.35
LG	40.78	40.78	44.19	277.18	56.79
CAT_GTR	**2.13**	7.22	7.29	110.79	16.01
CAT_F81	** − 3.48**	17.48	** − 985.18**	75.66	**4.48**

The table shows *Z-*scores for five PPAs, *Z* values < 5 are shown in bold. For each PPA test in this table, the observed heterogeneity, the posterior predictive mean heterogeneity and the SD around the mean are given in the Additional file 1: Table S3 and S4 available on Dryad

### Divergence time estimation and ancestral state reconstructions (ASR)

**Fig. 8:**
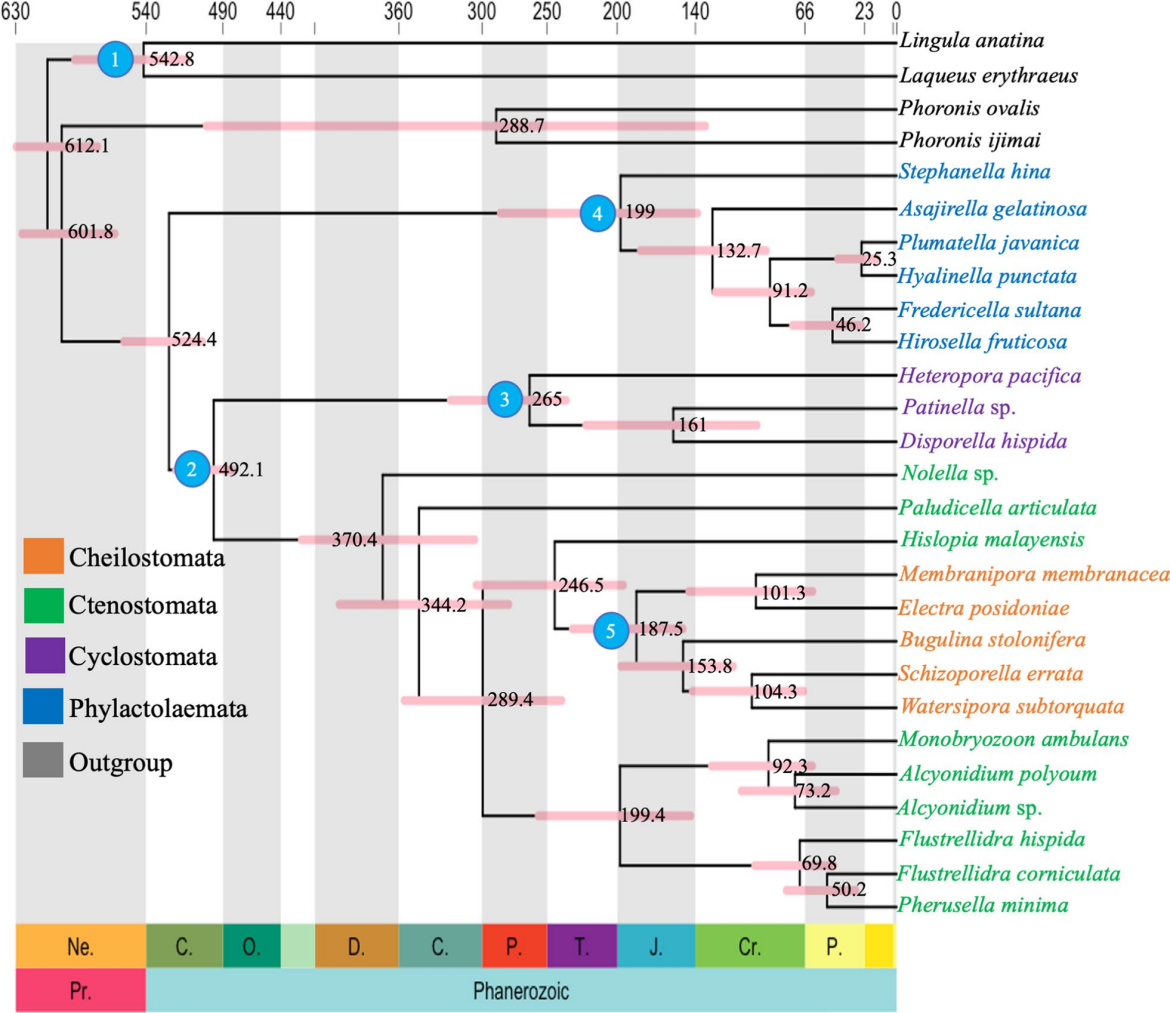
Time-calibrated phylogeny of Bryozoa using MCMCTree based on the complete data matrix with posterior distributions based on the truncated-Cauchy priors. A time scale in Ma is shown above the tree, with geographical periods labelled below the tree. Node bars represent 95% confidence intervals of age estimates and raw numbers for mean. Numbered circles represent nodes with fossil calibrations, corresponding to the numbers in Additional file 1: Table S5

We estimated divergence times using a relaxed independent rates molecular clock model with three different calibration strategies: truncated-Cauchy (Fig. 8), skew normal (Fig. S8), and uniform (Fig. S9); Additional file 1).

We focused on the divergence time of *M. ambulans.* All of our divergence time estimates showed that *M. ambulans* evolved from its most recent common ancestor in the Late Cretaceous at approximately 92.3 million years (Ma) with 95% credibility interval [CI], 60.4 to 130.3 Ma based on truncated-Cauchy analysis, at∼96.2 Ma (CI, 62.6–130.9 Ma) based on the skew normal analysis and at∼96.6 Ma (CI, 62.3–160.8 Ma) based on uniform analysis.

Our ASR analysis showed that a colonial lifestyle is ancestral within bryozoans and that the solitary lifestyle evolved independently in *M. ambulans* (Additional file 1: Fig. S10).

### Mitochondrial genome

**Fig. 9:**
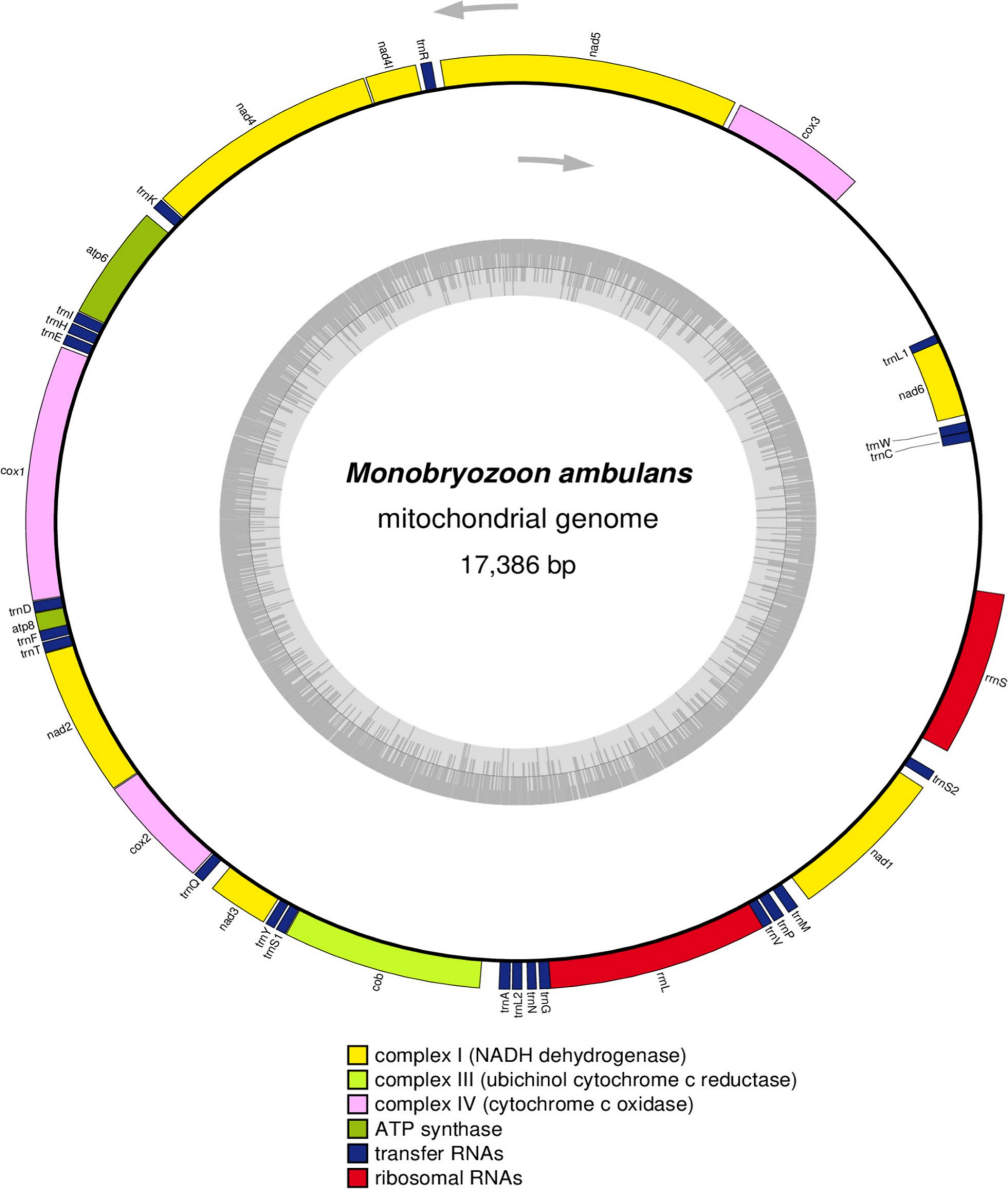
Circular gene map of the complete mitochondrial genome of *Monobryozoon ambulans*. The inner grey circles show the GC content. Different functional gene groups are color-coded. Grey arrows indicate the direction of transcription of the two DNA strands

The complete mitogenome of *M. ambulans* was assembled into a single contig 17,386 bp long. It consisted of 13 PCGs., two rRNAs and 22 tRNAs (Fig. 9). Our results showed that 12 PCGs, two rRNA and 19 tRNA genes are transcribed on the forward strand while only one PCG (*nad6*) and three tRNA genes (*trnC*, *trnL1* and *trnW*) are transcribed on the reverse strand (Fig. 9). The GC content for *M. ambulans* mitochondrial genome was 33.3%.

## Discussion

### Morphological characters

#### General morphology, appendages and solitary bryozoans

The general morphology of *Monobryoon ambulans* is similar to the original descriptions [8, 9], but contrary to the original report we found distinct septation between the proximal appendages and the remaining sac-shaped feeding zooid (autozooid). This implies that the appendages are not part of the zooid itself—in the form of so-called cystid appendages—but probably represent true polymorphs, termed kenozooids (see also [46] for bryozoan polymorphism). The appendages of *M. ambulans* also contain thin parietal muscles in the proximal area, which, in contrast to the original description, are not continuous with the main body cavity of the autozooid, but remain entirely within the appendage owing also to the cuticular septation first described in the current study.

Distinct glandular or adhesive properties of the distal part of the appendages, as previously proposed [8], could not be verified from our histological analyses. More live observations and ultrastructural analyses are necessary to confirm these observations.

Contrary to kenozooids, simple cystid appendages would lack any septation and have continuous cavities with the zooid, as e.g. found in arachnidiid and nolellid ctenostomes [5]. However, interzooidal septa are perforated by specific pore complexes showing a specific set of cells at the interface [47, 48], which could only be detected at the appendage carrying the bud.

Buds show a similar vesicle-like two-layered anlage of the polypide, as found in all other bryozoans (see [48–50]). In the encountered specimens of the current study, all buds were young and showed a similar appearance. Older buds were previously documented, also in association with a second young bud in formation. Such stages, however, only appear when the older bud is almost finished and ready to separate from the mother zooid [8, 9]. The entire process of appendage formation in budding stages would be an interesting aspect to study in the future. Gonads were not found in our specimens, but were previously observed in *M. ambulans* [10] and also *M. bulbosum* [11]. Oocytes appear to be large indicating yolky, lecithotrophic development in the genus.

We present the first confirmation of possible non-coloniality in monobryozoids, but together with observations on aethozoid ctenostomes, we follow our initial definition of single autozoids with thin, even polymorphic ones as solitary. This follows the observation of over 100 aethozoids which never showed more than one functional zooid and often lacked any polymorphic appendages altogether (Schwaha, pers. observation). In addition, kenozooidal appendages are most likely buds that separate from the mother animal in later stages ([6], Schwaha, pers. observation). We also consider the polymorphic appendages of monobryozoids as bud anlagen, of which, contrary to aethozoids, a high number is produced simultaneously, but only one of them develops into a new zooid, which will separate from the mother animal (see also [10]). Evidence for this hypothesis is also seen in the appendages of *M. bulbosum*, which start as thin finger-like extensions of the zooids to form bulbous appendages [11]. In the latter it has not been studied in detail at what point true septations form to the bulbous appendages. The formation of numerous buds in form of appendages seems to be an adaptation to maintain mobility and flexibility in sand sediments.

### Lophophore and digestive tract

The lophophore of *Monobryozoon ambulans* has 12 tentacles similar to the range of 13–14 previously mentioned by Remane [8] and is thus in the lower range among ctenostomes. The lowest number present in ctenostomes are eight tentacles as found in *M. bulbosum* [11] and in many victorellid or vesicularioidean ctenostomes. Alcyonidioideans rarely have 12 tentacles and tend to range from 16 to over 20 tentacles per zooid [51].

Ctenostome bryozoans show two basic gut configurations with either the anus being close to the vestibulum or the lophophoral base [52]. Incidentally, the distribution of these anal positions has proved to be of valuable systematic information. As previously indicated [52] this study firmly supports a vestibular position of the *Monobryozoon* anus, which again supports a closer affinity to alcyonidioidean bryozoans.

### ‘Ciliated grooves’

A very peculiar structure observed in *Monobryozoon ambulans* are so-called ciliated grooves [9, 10], which are vestibular folds supposedly carrying cilia with granular particles. As such, they were termed ‘ciliated organ’ or just ciliated depressions and suggested to be sensory (based on included particles) or excretory. Such vestibular grooves, or termed folds herein, were detected in our current analysis, but we failed to detect any sign of ciliation, which, however, would require electron microscopic studies to ascertain. Still, distinct granular material was observed in this study, but so far neither a sensory nor excretory function can be ascertained to this structure.

### Muscular system

Parietal muscles are typical of all gymnolaemates and consist of bundles traversing the body cavity [5, 53, 54]. In *M. ambulans* parietal muscles are exceptionally diffusedly arranged over many areas of the body wall. In most gymnolaemates, they are usually arranged as a series of regular pairs, except in alcyonidioidean ctenostomes that show a rather diffuse serial arrangement [54]. Thus, the diffuse arrangement of parietal muscles is a shared character of *Monobryozoon* and alcyonidioideans.

An orificial sphincter prominently lining the vestibular wall as observed in *Monobryozoon ambulans* is rare among ctenostome bryozoans, but found in alcyonidioideans [5, 54]. It was even considered apomorphic for the superfamily [5], but observations on multiporate alcyonidioideans have shown that they lack such a muscle [13, 55, 56]. Hence it could be apomorphic for Alcyonidiidae, including *Monobryozoon ambulans*.

Duplicature bands in the apertural region are part of the body plan of bryozoans [57]. Most ctenostomes have four and only occasionally more than that [13, 54, 57–59]. Five bands as found in *M. ambulans* was only reported in the alcyonidiid multiporate *Elzerina binderi* [13].

Besides tentacle muscles and more prominent foregut musculature, the remaining musculature of the gut seems absent or not stained in the current analyses. Although not completely understood, alcyonidioideans generally show only a few muscles on their guts in phalloidin stainings, contrary to other ctenostomes (see [5, 50, 56], and adds another possible shared character of *Monobryozoon* to alcyonidioideans.

### Molecular data

#### Phylogenetic analysis

We generated two new transcriptomes and combined them with publicly available transcriptomes of representative taxa of the main bryozoan clades: Cheilostomata, Ctenostomata, Cyclostomata and Phylactolaemata. We have assembled two datasets: the complete data matrix (including all 2,014 OGs) and the subsampled data matrix (including only the best 1500 OGs based on the nRCFV value). The analyses of these two data matrices were compared to assess the effects of removing genes with poor nRCFV scores on phylogenetic reconstruction.

Our phylogeny is consistent with previous molecular studies [23, 60, 61] providing strong support for the sister group relationships between Phylactolaemata and Myolaemata as well as the sister group relationships between Gymnolaemata and Stenolaemata. All ML analyses of both data matrices and the BI analysis of the subsampled data matrix strongly supported *M. ambulans* as the sister taxon to a clade comprising two *Alcyonidium* species. However, the phylogenetic position of *M. ambulans* in the BI analysis of the complete data matrix with CAT-F81 + G4 model conflicted with other analyses by placing *M. ambulans* as the sister taxon to *Alcyonidium polyoum*, but without significant support PP = 0.51). The phylogenetic placement of *M. ambulans* has been long debated, but was often considered uncertain [51, 62] or closely related to arachnidioideans [4, 63]. Alcyonidiid affinities were first proposed by d’Hondt [64, 65], which was corroborated by our phylogenetic analyses. The first bryozoan phylogenetic analysis including *M. ambulans* based on transcriptomic data supports this hypothesis. The placement of *M. ambulans* based on BI analysis of the complete data matrix with CAT-F81 + G4 model might be due to the poor convergence of this analysis. Convergence in the posterior distribution of parameters is a crucial aspect in evaluating the quality of any BI analysis and several studies have cautioned against relying on results that fail to converge, for obvious reasons [66–69].

Alcyonidioidea was previously shown to represent the sister taxon to all other gymnolaemates [61]. This placement is not supported in our phylogeny, but instead we recovered *Nolella* as the sister taxon to the remaining gymnolaemates in all phylogenetic analyses, except for the BI analysis with the CAT-GTR + G4 model of the subsampled data matrix, while *Paludicella* was recovered as the sister taxon to the remaining gymnolaemates. This contradictory results about the phylogenetic position of the early branching species within gymnolaemates might be due to the poor taxon sampling of these species, particularly *Nolella*, which includes approximately 18 recent species [5]. Another possible reason for the different branching pattern of *Nolella* and *Paludicella* is the choice of the model of sequence evolution in the BI analysis. Here, in the same dataset (the subsampled data matrix), the CAT-F81 + G4 analysis yielded the same branching order concerning these particular taxa as the ML analyses, while the CAT-GTR + G4 analysis gave different tree topologies suggesting that at least one category of models is subject to a systematic error.

Overall tree topologies are largely congruent between the complete and the subsampled datasets, with the exception of some nodes notated above. In addition, we found that removing compositionally heterogeneous OGs from the data matrix has improved the convergence statistics and the model fitness of the BI analyses under PPA (particularly PPA-MEAN and PPA-MAX) relative to the complete dataset. Compositional heterogeneity has been suggested as a significant factor leading to systematic errors in phylogenetic analyses [70, 71]. Our PPA results showed that the CAT-F81 + G4 model explains the data much better than the site-homogeneous LG and GTR models and surprisingly even better than the CAT-GTR + G4 model. However, it has been suggested that the CAT-F81 model can cause systematic error in analyses of empirical data [72].

In line with the reconstructed phylogeny, we inferred coloniality as the ancestral state of bryozoans and that a solitary or pseudo-colonial lifestyle evolved independently in the Late Cretaceous in the ancestor of *M. ambulans*. Particularly in the Late Cretaceous a massive diversification of cyclostome and cheilostome bryozoans is evident in the fossil record. This diversification is even more pronounced in cheilostomes once brooding mechanisms appeared in the fossil record [73]. Competition for substrates and food is a major evolutionary vector in bryozoans. Possibly, the high competition of other bryozoans clades led ctenostome bryozoans such as monobryozoids to find new, atypical habitats such as sand bottoms.

The solitary lifestyle probably evolved independently in aethozoid ctenostomes [6]. So far, only morphological data are available for aethozoids, which already show strong contrasts to monobryozoids in many aspects (see also [7, 74]). Sequence data of any kind is still missing for any species, however.

In this regard, we would like to emphasize the importance of combined analyses using morphological and molecular methods to assess systematic positions of disputed taxa such as *Monobryozoon*. Although, often difficult, the establishment of shared morphological characters as seen in *Monobryozoon* and alcyonidiids can yield much information on morphological character evolution within systematic clades.

Ultimately, the phylogenetic position of *Monobryzoon* within ctenostomes remains little understood from an evolutionary perspective. Contrary to aethozoids, which often have only widely connected zooids, alcyonidiids always form very tight, dense and often large colonies. How such a solitary lifestyle evolved from such ancestral forms remains difficult to assess, but there remain many bryozoans to be discovered that could tell another tale.

### Mitochondrial genome

The mitochondrial genome of *M. ambulans* shows typical characteristics of metazoan mitogenomes, consisting of 13 PCGs., two rRNAs and 22 tRNAs. The size of *M. ambulans* mitochondrial genomes is 17,386 bp in length which falls within the size range of other studied mitogenomes of gymnolaemate bryozoans that range from 13,026 bp in *F. hispida* [75] to 23,057 bp *Exechonella vieirai* [76]. The GC content of *M. ambulans* mitogenome was 33.3% which is somewhat higher than the GC content of *B. neritina* (30%) [44] (Jang et al. 2009) and lower than *F. hispida* (41.6%) [75].

## Conclusion

This is the first sighting of *M. ambulans* since 1971, and from its type locality since 1938. This is also the first study to combine morphological and molecular data of this enigmatic species. Morphological aspects show that *Monobryozoon* is pseudo-colonial and has kenozooidal, but most likely transitory polymorphs attached to it. While this basic body organization of *M. ambulans* shows similarities to aethozoid ctenostomes, details of its gut structure and muscular organization strongly differ, which probably indicates an independent evolution of a solitary lifestyle in these bryozoan families. Both morphology and molecular data confirm that monobryozoans are closely allied to alcyonidiid ctenostomes. Future research should try to gain more knowledge into the ecology and reproduction of these animals, but also acquire sequence data for aethozoids to test whether they independently evolved a solitary lifestyle.

### Supplementary Information

**Additional file 1: Table S1**. Details of the specimens, collection localities, GenBank Sequence Read Archive (SRA) accession numbers and sources of publicly available sequences. **Table S2**. Tests of compositional homogeneity determine whether models adequately represented the compositional variation of the data for both data matrices. **Table S3**. Detailed results of PPA analyses related to Table 1. This table illustrates the empirical heterogeneity observed directly from the data, the average posterior predictive mean and the dispersion around the mean for the mean amino for the five PPA statistics of the complete dataset. **Table S4**. Detailed results of PPA analyses related to Table 1. This table illustrates the empirical heterogeneity observed directly from the data, the average posterior predictive mean and the dispersion around the mean for the mean amino for the five PPA statistics of the subsampled dataset. **Table S5.** Fossil calibration nodes used. Each of the nodes used in calibration have the same number in Fig. 7. The second column gives the input used in MCMCTree for the Cauchy “L”, skew normal “SN” and uniform “B” prior age distributions. **Table S6**. Summary statistics of the transcriptome assembly for *Monobryozoon ambulans*. **Table S7**. Number of unique and total functional annotation of the *Monobryozoon ambulans* transcriptome using Trinotate pipeline. **Figure S1.** Maximum likelihood phylogeny of Bryozoa based on the complete data matrix, including 422,961 AAs from 2,014 OGs using unpartitioned analysis with PMSF model. All nodes are supported by 100 ultrafast bootstraps. The scale bar represents 1 substitutional change per 100 AAs. **Figure S2.** Bayesian inference tree of Bryozoa based on the complete data matrix, including 422,961 AAs from 2,014 OGs with CAT-F81 + G4 model. Bayesian posterior probabilities are only shown for nodes that are not maximally supported. The scale bar represents 1 substitutional change per 100 AAs. **Figure S3.** Bayesian inference tree of Bryozoa based on the complete data matrix, including 422,961 AAs from 2,014 OGs with CAT-GTR + G4 model. Bayesian posterior probabilities are only shown for nodes that are not maximally supported. The scale bar represents 1 substitutional change per 100 AAs. **Figure S4**. Maximum likelihood phylogeny of Bryozoa based on the subsampled data matrix, including 310,190 AAs from 1500 OGs using partitioned analysis. Values on some nodes represent ML ultrafast bootstrap support and only shown for nodes that are not maximally supported by all analyses. The scale bar represents 1 substitutional change per 100 AAs. **Figure S5**. Maximum likelihood phylogeny of Bryozoa based on the subsampled data matrix, including 310,190 AAs from 1500 OGs using unpartitioned analysis with PMSF model. All nodes are supported by 100 ultrafast bootstraps. The scale bar represents 1 substitutional change per 100 AAs. **Figure S6**. Bayesian inference tree of Bryozoa based on the subsampled data matrix, including 310,190 AAs from 1500 OGs with CAT-F81 + G4 model. Bayesian posterior probabilities are only shown for nodes that are not maximally supported. The scale bar represents 1 substitutional change per 100 AAs. **Figure S7**. Bayesian inference tree of Bryozoa based on the subsampled data matrix, including 310,190 AAs from 1500 OGs with CAT-GTR + G4 model. Bayesian posterior probabilities are only shown for nodes that are not maximally supported. The scale bar represents 1 substitutional change per 100 AAs. **Figure S8**. Time-calibrated phylogeny of Bryozoa using MCMCTree based on the complete data matrix with posterior distributions based on the skew normal priors. A time scale in Ma is shown above the tree, with geographical periods labelled below the tree. Node bars represent 95% confidence intervals of age estimates and raw numbers for mean. **Figure S9**. Time-calibrated phylogeny of Bryozoa using MCMCTree based on the complete data matrix with posterior distributions based on the uniform priors. A time scale in Ma is shown above the tree, with geographical periods labelled below the tree. Node bars represent 95% confidence intervals of age estimates and raw numbers for mean. **Figure S10**. Ancestral state reconstruction for the lifestyles in bryozoans. The pie area indicates the likelihood of character state at each node. **Figure S11**. Convergence plots for the divergence time analyses showing the relationship between the posterior mean of the two runs of each calibration strategy (A Cauchy, B skew normal and C uniform). **Figure S12**. Top 20 species taxonomic distribution on the basis of BLASTX and BLASTP hits of *Monobryozoon ambulans* transcriptome against UniRef90 database. **Figure S13**. GO functional annotation of *Monobryozoon ambulans* transcriptome. Orange represents cellular component, blue represents biological process, and green represents molecular function. The Y-axis represents distribution of the top 15 GO terms of each category, the X-axis the number of transcripts. Pie-charts showing the percentage of three GO categories. **Figure S14**. Classification of eggNOG annotations of the *Monobryozoon ambulans* transcriptome. The capital letters on the Y-axis represent different eggNOG categories. X-axis shows the number of transcripts in each eggNOG category.

## Data Availability

Data available from the figshare Digital Repository: 10.6084/m9.figshare.25151126.The sequence data generated in this study are available from NCBI under BioProject accession PRJNA1002113.

## References

[1] Bleidorn C (2019). Recent progress in reconstructing lophotrochozoan (spiralian) phylogeny. Org Divers Evol.

[2] Ryland JS (1970). Bryozoans.

[3] Schwaha T, Ostrovsky AN, Wanninger A (2020). Key novelties in the evolution of aquatic colonial phylum Bryozoa: evidence from soft body morphology. Biol Rev.

[4] Bock P, Gordon DP (2013). Phylum Bryozoa Ehrenberg, 1831. Zootaxa.

[5] Schwaha T, Schwaha T (2020). Ctenostomata. Handbook of zoology Bryozoa.

[6] Schwaha T, Edgcomb VP, Bernhard JM, Todaro MA (2019). *Aethozooides uraniae*, a new deep sea genus and species of solitary bryozoan from the Mediterranean with a revision of the Aethozoidae. Mar Biodivers.

[7] Schwaha T, Ott JA, Schmidt-Rhaesa A (2020). 17 Bryozoa. Guide to the identification of Marine Meiofauna.

[8] Remane A (1936). Monobryozoon ambulans n. gen., n. sp., ein eigenartiges Bryozoon des Meeressandes. Zool Anz.

[9] Remane A (1938). Ergänzende Mitteilungen über *Monobryozoon ambulans* Remane. Kieler Meeresforsch.

[10] Gray JS (1971). Occurrence of the aberrant bryozoan *Monobryozoon ambulans* Remane, off the Yorkshire coast. J Nat Hist.

[11] Ott JA. *Monobryozoon bulbosum* n. sp., a New solitary interstitial bryozoan from the west Atlantic coast. Cah Biol Mar. 1972;13:421–428.

[12] d’Hondt J-L, Hayward PL (1981). Nouvelles recoltes de Bryozoaires Cténostomes bathyaux et abyssaux. Cah Biol Mar.

[13] Schwaha T (2021). Morphology of ctenostome bryozoans. 3. Elzerina, Flustrellidra, Bockiella. J Morphol.

[14] Schindelin J, Arganda-Carreras I, Frise E, Kaynig V, Longair M, Pietzsch T, Preibisch S, Rueden C, Saalfeld S, Schmid B (2012). Fiji: an open-source platform for biological-image analysis. Nat Methods.

[15] Ruthensteiner B (2008). Soft Part 3D visualization by serial sectioning and computer reconstruction. Zoosymposia.

[16] Bolger AM, Lohse M, Usadel B (2014). Trimmomatic: a flexible trimmer for Illumina sequence data. Bioinformatics.

[17] Haas BJ, Papanicolaou A, Yassour M, Grabherr M, Blood PD, Bowden J, Couger MB, Eccles D, Li B, Lieber M (2013). De novo transcript sequence reconstruction from RNA-seq using the Trinity platform for reference generation and analysis. Nat Protoc.

[18] Khalturin K, Shunatova N, Shchenkov S, Sasakura Y, Kawamitsu M, Satoh N (2022). Polyzoa is back: the effect of complete gene sets on the placement of Ectoprocta and Entoprocta. Sci Adv.

[19] Fu L, Niu B, Zhu Z, Wu S, Li W (2012). CD-HIT: accelerated for clustering the next-generation sequencing data. Bioinformatics.

[20] Simão FA, Waterhouse RM, Ioannidis P, Kriventseva EV, Zdobnov EM (2015). BUSCO: assessing genome assembly and annotation completeness with single-copy orthologs. Bioinformatics.

[21] Emms DM, Kelly S (2019). OrthoFinder: phylogenetic orthology inference for comparative genomics. Genome Biol.

[22] Kocot KM, Struck TH, Merkel J, Waits DS, Todt C, Brannock PM, Weese DA, Cannon JT, Moroz LL, Lieb B (2017). Phylogenomics of Lophotrochozoa with consideration of systematic error. Syst Biol.

[23] Saadi AJ, Bibermair J, Kocot KM, Roberts NG, Hirose M, Calcino A, Baranyi C, Chaichana R, Wood TS, Schwaha T (2022). Phylogenomics reveals deep relationships and diversification within phylactolaemate bryozoans. Proc R Soc B Biol Sci.

[24] Katoh K, Standley DM (2013). MAFFT multiple sequence alignment software version 7: improvements in performance and usability. Mol Biol Evol.

[25] Di Franco A, Poujol R, Baurain D, Philippe H (2019). Evaluating the usefulness of alignment filtering methods to reduce the impact of errors on evolutionary inferences. BMC Evol Biol.

[26] Criscuolo A, Gribaldo S (2010). BMGE (Block Mapping and Gathering with Entropy): a new software for selection of phylogenetic informative regions from multiple sequence alignments. BMC Evol Biol.

[27] Price MN, Dehal PS, Arkin AP (2009). FastTree: computing large minimum evolution trees with profiles instead of a distance matrix. Mol Biol Evol.

[28] Fleming JF, Struck TH (2023). nRCFV: a new, dataset-size-independent metric to quantify compositional heterogeneity in nucleotide and amino acid datasets. BMC Bioinf.

[29] Kück P, Meusemann K (2010). FASconCAT: convenient handling of data matrices. Mol Phylogenet Evol.

[30] Minh BQ, Schmidt HA, Chernomor O, Schrempf D, Woodhams MD, von Haeseler A, Lanfear R (2020). IQ-TREE 2: new models and efficient methods for phylogenetic inference in the genomic era. Mol Biol Evol.

[31] Kalyaanamoorthy S, Minh BQ, Wong TKF, von Haeseler A, Jermiin LS (2017). ModelFinder: fast model selection for accurate phylogenetic estimates. Nat Methods.

[32] Wang H-C, Minh BQ, Susko E, Roger AJ (2017). Modeling site heterogeneity with posterior mean site frequency profiles accelerates accurate phylogenomic estimation. Syst Biol.

[33] Lartillot N, Rodrigue N, Stubbs D, Richer J (2013). PhyloBayes MPI: phylogenetic reconstruction with infinite mixtures of profiles in a parallel environment. Syst Biol.

[34] Nascimento FF, Reis Md, Yang Z (2017). A biologist’s guide to Bayesian phylogenetic analysis. Nat Ecol Evol.

[35] Feuda R, Dohrmann M, Pett W, Philippe H, Rota-Stabelli O, Lartillot N, Wörheide G, Pisani D (2017). Improved modeling of compositional heterogeneity supports sponges as sister to all other animals. Curr Biol.

[36] Blanquart S, Lartillot N (2008). A site- and time-heterogeneous model of amino acid replacement. Mol Biol Evol.

[37] Lartillot N. The Bayesian approach to molecular phylogeny. In: Scornavacca C, Delsuc F, Galtier N (eds) Phylogenetics in the Genomic Era (no commercial publisher, open access book); 2020: 1.4:1–1.4:17.

[38] Yang Z (2007). PAML 4: phylogenetic analysis by maximum likelihood. Mol Biol Evol.

[39] Puttick MN (2019). MCMCtreeR: functions to prepare MCMCtree analyses and visualize posterior ages on trees. Bioinformatics.

[40] Maddison WP, Maddison DR. Mesquite: a modular system for evolutionary analysis. 2021. Version 3.70. https://mesquiteproject.org/.

[41] Bankevich A, Nurk S, Antipov D, Gurevich AA, Dvorkin M, Kulikov AS, Lesin VM, Nikolenko SI, Pham S, Prjibelski AD, Pyshkin AV, Sirotkin AV, Vyahhi N, Tesler G, Alekseyev MA, Pevzner PA (2012). SPAdes: a new genome assembly algorithm and its applications to single-cell sequencing. J Comput Biol.

[42] Altschul SF, Gish W, Miller W, Myers EW, Lipman DJ (1990). Basic local alignment search tool. J Mol Biol.

[43] Donath A, Jühling F, Al-Arab M, Bernhart SH, Reinhardt F, Stadler PF, Middendorf M, Bernt M (2019). Improved annotation of protein-coding genes boundaries in metazoan mitochondrial genomes. Nucleic Acids Res.

[44] Jang KH, Hwang UW (2009). Complete mitochondrial genome of *Bugula neritina* (Bryozoa, Gymnolaemata, Cheilostomata): phylogenetic position of Bryozoa and phylogeny of lophophorates within the Lophotrochozoa. BMC Genomics.

[45] Greiner S, Lehwark P, Bock R (2019). OrganellarGenomeDRAW (OGDRAW) version 1.3.1: expanded toolkit for the graphical visualization of organellar genomes. Nucleic Acids Res.

[46] Schack CR, Gordon DP, Ryan KG (2019). Modularity is the mother of invention: a review of polymorphism in bryozoans. Biol Rev.

[47] Bobin G, Woollacott RM, Zimmer RL (1977). Interzooecial communications and the funicular system. Biology of Bryozoans.

[48] Mukai H, Terakado K, Reed CG, Harrison FW, Woollacott RM (1997). Bryozoa. Microscopic anatomy of invertebrates.

[49] Lutaud G, Robinson RA (1983). Autozooid morphogenesis in anascan cheilostomates. Treatise on invertebrates Palaeontology Part G: Bryozoa (revised).

[50] Schwaha T, Wood TS, Wanninger A (2011). Myoanatomy and serotonergic nervous system of the ctenostome Hislopia malayensis: evolutionary trends in bodyplan patterning of Ectoprocta. Front Zool.

[51] D’Hondt JL (1983). Tabular keys for identification of the recent Ctenostomatous Bryozoa. Mémoires de L’Institut Océanographique, Monaco.

[52] Schwaha T (2020). O anus, where art thou? An investigation of ctenostome bryozoans. J Morphol.

[53] Cheetham AH, Cook PL, Robinson RA (1983). General features of the class gymnolaemata. Treatise on invertebrate paleontology Part G: Bryozoa.

[54] Schwaha T, Wanninger A (2018). Unity in diversity: a survey of muscular systems of ctenostome Gymnolaemata (Lophotrochozoa, Bryozoa). Front Zool.

[55] Schwaha T, Winston JE, Gordon DP (2022). Morphology of ctenostome bryozoans: 5. Sundanella, with description of a new species from the Western Atlantic and the Multiporata concept. J Morphol.

[56] Decker S, Wanninger A, Schwaha T (2020). Morphology and life cycle of an epiphytic pherusellid ctenostome bryozoan from the Mediterranean Sea. Org Divers Evol.

[57] Schwaha T, Schwaha T (2020). Morphology of bryozoans. Handbook of zoology: Bryozoa.

[58] Schwaha T, De Blauwe H (2020). Morphology of ctenostome bryozoans: 1. *Arachnidium fibrosum*. J Morphol.

[59] Schwaha T, Grischenko AV, Melnik VP (2020). Morphology of ctenostome bryozoans: 2. Haywardozoon pacificum, with implications of the phylogenetic position of the genus. J Morphol.

[60] Orr RJS, Di Martino E, Ramsfjell MH, Gordon DP, Berning B, Chowdhury I, Craig S, Cumming RL, Figuerola B, Florence W (2022). Paleozoic origins of cheilostome bryozoans and their parental care inferred by a new genome-skimmed phylogeny. Sci Adv.

[61] Waeschenbach A, Taylor PD, Littlewood DTJ (2012). A molecular phylogeny of bryozoans. Mol Phylogenet Evol.

[62] Todd JA. The central role of ctenostomes in bryozoan phylogeny. In: Proceedings of the 11th International Bryozoology Association Conference. Edited by Herrera Cubilla A, Jackson JBC. Balboa: Smithsonian Tropical Research Institute; 2000: 104–135.

[63] Reverter-Gil O, Souto J, Fernández Pulpeiro E. Fauna Iberica. Vol 43. Bryozoa 1. Ctenostomata. Madrid: Museo Nacional de Ciencias Naturales; 2016.

[64] D’Hondt JL (1986). Etat de connaissances sur las position phylogenetique et l’evolution des bryozoaires. Boll Zool.

[65] D’Hondt JL (1997). La classification actuelle des bryozoaires eurystomes. Bull Soc Zool Fr.

[66] Nylander JAA, Wilgenbusch JC, Warren DL, Swofford DL (2007). AWTY (are we there yet?): a system for graphical exploration of MCMC convergence in Bayesian phylogenetics. Bioinformatics.

[67] Rodrigue N, Lartillot N (2013). Site-heterogeneous mutation-selection models within the PhyloBayes-MPI package. Bioinformatics.

[68] Steppan SJ, Schenk JJ (2017). Muroid rodent phylogenetics: 900-species tree reveals increasing diversification rates. PLoS ONE.

[69] Harrington SM, Wishingrad V, Thomson RC (2020). Properties of Markov chain Monte Carlo performance across many empirical alignments. Mol Biol Evol.

[70] Zhong M, Hansen B, Nesnidal M, Golombek A, Halanych KM, Struck TH (2011). Detecting the symplesiomorphy trap: a multigene phylogenetic analysis of terebelliform annelids. BMC Evol Biol.

[71] Nesnidal MP, Helmkampf M, Meyer A, Witek A, Bruchhaus I, Ebersberger I, Hankeln T, Lieb B, Struck TH, Hausdorf B. New phylogenomic data support the monophyly of Lophophorata and an Ectoproct-Phoronid clade and indicate that Polyzoa and Kryptrochozoa are caused by systematic bias. BMC Evol Biol 2013;13. 10.1186/1471-2148-13-253 PMC4225663 24238092

[72] Whelan NV, Halanych KM (2016). Who let the CAT out of the bag? Accurately dealing with substitutional heterogeneity in phylogenomic analyses. Syst Biol.

[73] Taylor PD (2020). Bryozoan Paleobiology.

[74] Schwaha T, Zeppilli D, González-Casarrubios A, Cepeda D (2024). The first deep-sea ctenostome bryozoan from the Indian Ocean: Aethozoon flavum sp. nov. Mar Biodivers.

[75] Waeschenbach A, Telford MJ, Porter JS, Littlewood DTJ (2006). The complete mitochondrial genome of *Flustrellidra hispida* and the phylogenetic position of Bryozoa among the Metazoa. Mol Phylogenet Evol.

[76] Jenkins HL, Graham R, Porter JS, Vieira LM, Almeida ACS, Hall A, O’Dea A, Coppard SE, Waeschenbach A (2023). Unprecedented frequency of mitochondrial introns in colonial bilaterians. Sci Rep.

