# Supplementary material for: Rediscovering the unusual, solitary bryozoan Monobryozoon ambulans Remane, 1936: first molecular and new morphological data clarify its phylogenetic position
Source: Front Zool. 2024 Mar 5;21:5. doi: 10.1186/s12983-024-00527-1 (PMC10913646; doi:10.1186/s12983-024-00527-1)
Supplement: Supplementary file 1 — Additional file 1: Table S1. Details of the specimens, collection localities, GenBank Sequence Read Archive (SRA) accession numbers and sources of publicly available sequences. Table S2. Tests of compositional homogeneity determine whether models adequately represented the compositional variation of the data for both data matrices. Table S3. Detailed results of PPA analyses related to Table 1. This table illustrates the empirical heterogeneity observed directly from the data, the average posterior predictive mean and the dispersion around the mean for the mean amino for the five PPA statistics of the complete dataset. Table S4. Detailed results of PPA analyses related to Table 1. This table illustrates the empirical heterogeneity observed directly from the data, the average posterior predictive mean and the dispersion around the mean for the mean amino for the five PPA statistics of the subsampled dataset. Table S5. Fossil calibration nodes used. Each of the nodes used in calibration have the same number in Fig. 7. The second column gives the input used in MCMCTree for the Cauchy “L”, skew normal “SN” and uniform “B” prior age distributions. Table S6. Summary statistics of the transcriptome assembly for Monobryozoon ambulans. Table S7. Number of unique and total functional annotation of the Monobryozoon ambulans transcriptome using Trinotate pipeline. Figure S1. Maximum likelihood phylogeny of Bryozoa based on the complete data matrix, including 422,961 AAs from 2,014 OGs using unpartitioned analysis with PMSF model. All nodes are supported by 100 ultrafast bootstraps. The scale bar represents 1 substitutional change per 100 AAs. Figure S2. Bayesian inference tree of Bryozoa based on the complete data matrix, including 422,961 AAs from 2,014 OGs with CAT-F81 + G4 model. Bayesian posterior probabilities are only shown for nodes that are not maximally supported. The scale bar represents 1 substitutional change per 100 AAs. Figure S3. Bayesian inference tree of Bryozoa [file 12983_2024_527_MOESM1_ESM.docx]

**Supplementary Materials for**

**Rediscovering the unusual, solitary bryozoan *Monobryozoon ambulans*, Remane 1936: First molecular and new morphological data clarify its phylogenetic position**

Thomas Schwaha^1$^, Sebastian H. Decker^1^, Christian Baranyi^1^, Ahmed J. Saadi^1^

^1^ University of Vienna, Department of Evolutionary Biology, Schlachthausgasse 43, 1030 Vienna, Austria

**ORCID**:

TS: 0000-0003-0526-6791

SHD: 0000-0001-9029-8717

CB: 0000-0003-3118-2145

AJS: 0000-0002-5113-0441

$ corresponding author

Dep. Evolutionary Biology, Schlachthausgasse 43, 1030 Vienna, Austria, [thomas.schwaha@univie.ac.at](mailto:thomas.schwaha@univie.ac.at)

Running Title: *Monobryozoon ambulans*

**Methods**

1. **Functional Annotation of *Monobryozoon ambulans* Transcriptome**

The Trinity transcriptome of *M. ambulans* was functionally annotated using Trinotate pipeline v3.1.1 with a cutoff E-value of 10^-5^ (<https://github.com/Trinotate/Trinotate.github.io>; last accessed July 29, 2021). Trinity transcriptome and transdecoder protein sequence files were searched for sequences homology using BLASTX and BLASTP (NCBI- blast v2.12.0+) [1] against the UniProt/SwissProt and UniRef90 databases, respectively. The assembled transcripts were also searched against Kyoto Encyclopedia of Genes and Genomes (KEGG), the evolutionary genealogy of genes: Non-supervised Orthologous Groups (EggNog), and Gene Ontology (GO) annotation databases. Furthermore, assembled transcripts were searched for potential ribosomal RNA using RNAmmer (v1.2) [2]. The transdecoder protein sequence file, was also searched for protein domains identification with HMMER v3.3 [3] using the Pfam domain database, potential signal peptides identification with SignalP v4.1 [4] and prediction of transmembrane helices with TMHMM v2.0c [5]. Finally, the annotations of the transcriptome were loaded into an SQLite database and reported in a tab-delimited file. The trinotateR package (https://github.com/cstubben/trinotateR; last accessed July 29, 2021) was used to summarize the results of the transcriptome annotations.

1. ***Divergence Time Estimation***

**Choice of Fossil Calibration Pointes**

We applied a conservative age constraint on the root of the input tree, utilizing a soft minimum age of 529 millions of years (Ma) based on the oldest brachiopod fossil which is known from Cambrian Stage 2 [6, 7] and a hard maximum age of 636.1 Ma, based on the origin of Spiralia / Lophotrochozoa [8].

The following age estimates of five carefully selected fossils were used to calibrate nodes of the input tree: 1) to calibrate the split between *Lingula anatina* and *Laqueus erythraeus,* a minimum age of 509 Ma was used based on the first fossil evidence of the brachiopod *Lingulella waptaensis* from middle Cambrian Burgess Shale [9]; 2) to calibrate the split between cyclostomes and cheilostomes+ ctenostomes, a minimum age of 478.6 Ma was used based on the oldest known fossil cyclostome (*Wolinella baltica* [10]). This fossil has been assigned to the Volkhov Stage of the Early Ordovician; 3) for the stem of cyclostomes, a minimum age of 237 Ma was used based on the earliest fossil evidence of cyclostome possessing brood chambers. *Reptomultisparsa hybensis* represents the earliest cyclostome with brood chambers which is known from the Late Triassic and occur in the Rhaetian of the Nezhne-Tatry Mountains, Czech Republic [11]; 4) to calibrate the stem of phylactolaemates, a minimum age of 163 Ma was used based on the first appearance of *Stephanella continentalis* Vinogradov in the Upper Jurassic of Russia [12]; 5) finally, for the stem of cheilostomes, a minimum age of 150 was used based on the oldest fossil record of cheilostomes (*Pyroporopsis portlandensis* of Electridae) which is known from the Late Jurassic Tithonian time of southern England [13]. Fossil calibration nodes used are giving in Table S5.

**MCMCTree Settings**

To make the divergence time analysis computationally feasible, the best-fitting model was first estimated for the complete data matrix (unpartitioned) using ModelFinder (part of IQ-TREE2 v2.1.2) [14] in which the model options were limited to only those that are available in MCMCTree (Dayhoff, DCMut, JTT, JTTDCMut, LG and WAG) with a maximum of five rate categories. The LG substitution model was chosen as the best model based on all three information criteria (AIC, AICc, and BIC), and it was subsequently used for dating analyses.

To specify the prior of rates, the independent rates model (clock=2) was used with approximate likelihood. For each of three analyses, two MCMC were conducted, each for 60.2 million iterations, with a burn-in of 200,000, sampling frequency of 6,000 and sample size of 10,000. This ensured that Effective Sample Size (ESS) for each parameter reached a sufficient size (ESS>200), as commonly recommended for MCMC-based Bayesian phylogenetic inference (Nascimento et al., 2017). The results of the two MCMC runs were evaluated in Tracer V1.7.2 [15] to check for convergence. Additionally, each MCMC run was examined for convergence by plotting posterior means of the two independent runs against each other in R (Fig. S11). Time priors were calculated by running MCMCTree without the sequence alignment (usedata=0) and were subsequently compared with the posterior times to ensure that the posterior density is contained within the prior density. Finally, the time-calibrated phylogeny was visualized using the R package MCMCtreeR [16].

**Results and discussion**

- - - 1. **De novo Assembly and Functional Annotation of *Monobryozoon ambulans***

In this study, we provide the first transcriptome of *M. ambulans* with functional annotations. An overview of the sequencing and assembly statistics of *M. ambulans* is shown in Table S6. The final assembly generated 102,642 transcripts with 23,079 contigs larger than 1000 Pb, N50 value of 1,197 and average GC percent content of 41%. Among the assembled transcripts, 36,428 were translated to putative proteins of which 22,693 were non-redundant proteins. BUSCO assessment of *M. ambulans* transcriptome with the Metazoa Odb10 database showed a reasonable level of completeness (74.8%) with a relatively high percentage of fragmented genes (13.6%) which are also classified as a positive match (partially recovered genes) [17].

The functional annotation of the *M. ambulans* transcriptome showed that among 102,642 assembled transcripts, 42,863 had unique BLASTX hits in the UniRef90 database and 39,842 had unique BLASTX hits in the UniProt/SwissProt database. Likewise, BLASTP annotation against Uniref90 and UniProt/SwissProt databases found 26,172 and 18,880 unique proteins, respectively (Trinotate annotation results are summarized in Table S7). Considering the annotation of the unique coding sequence regions in the *M. ambulans* transcriptome, a relatively high proportion of proteins have shown sequence homology against the UniRef90 and UniProt/SwissProt databases, which provides another validation of the quality of the transcriptome assembly. The BLAST top annotation hits distribution showed that the highest number of proteins and transcripts were from the bryozoan *Bugula neritina* followed by the brachiopod *Lingula unguis* (Fig. S12). This confirms that *M. ambulans* belongs to bryozoans, consistent with the recent phylogenetic analysis by Laumer et al. [18] which showed brachiopod as the sister clade of phoronids + bryozoans. The remaining BLAST hits exhibited similarities to other metazoan species in the majority of cases.

Furthermore, annotation results recovered 32,029 “GO terms” of which 11,315 were unique. The GO terms were assigned into three categories including biological process (46%), cellular compartment (31%) and molecular function (23%) as shown in Fig. S13. Under the biological process category, “ATP binding” represented the highest number of transcripts (4612), among the cellular component category, “cytoplasm” was the dominant subcategory with 9228 transcripts, and under the molecular function category, “transcription, DNAtemplated” had the highest number of transcripts (2397). A similar pattern of representation for the three main categories of “GO terms” has been found in the transcriptome annotation of the freshwater bryozoan *Fredericella sultana* [19].

The number of unique and total functional annotations of proteins against other databases (KEGG, SignalP and TmHMM) is shown in Table S7. Additionally, the number of unique genes, transcripts and proteins with Pfam annotation was 15,122, 26,996 and 27,507, respectively. The EggNOG classification of *M. ambulans* genes showed that 4,172 unique genes were identified in the EggNOG database and classified into 22 categories as shown in Fig. S14. The most abundant category was “Posttranslational modifications, protein turnover, chaperones” followed by “Intracellular trafficking, secretion, and vesicular transport”.

**Table S1**. Details of the specimens, collection localities, GenBank Sequence Read Archive (SRA) accession numbers and sources of publicly available sequences.

| **Family** | **Species** | **Accession number and download source** | **Reference** |
| --- | --- | --- | --- |
| **Phylactolaemata** | | | |
| Fredericellidae | *Fredericella sultana* Blumenbach, 1779 | NCBI, SRA, SAMN25648380 | Saadi et al. [20] |
| Lophopodidae | *Asajirella gelatinosa* 1 (Oka, 1891) | NCBI, SRA, SAMN25648383 | Saadi et al. [20] |
| Plumatellidae | *Hyalinella punctata* Vorstman, 1928 | NCBI, SRA, SAMN25648388 | Saadi et al. [20] |
|  | *Hirosella fruticosa* Allmann, 1844 | NCBI, SRA, SAMN25648393 | Saadi et al. [20] |
|  | *Plumatella javanica* Kraepelin, 1906 | NCBI, SRA, SAMN25648394 | Saadi et al. [20] |
| Stephanellidae | *Stephanella* cf. *hina* Oka, 1908 | NCBI, SRA, SAMN25648400 | Saadi et al. [20] |
| **Stenolaemata**  **Cyclostomata** | | | |
| Heteroporidae | *Heteropora pacifica* Borg, 1933 | NCBI, SRA, SRR9681687 | Laumer et al. [18] |
| Lichenoporidae | *Disporella hispida* (Fleming, 1828) | NCBI, SRA, SAMN25648401 | Saadi et al. [20] |
| Lichenoporidae | *Patinella* sp. | NCBI, SRA, SAMN25648402 | Saadi et al. [20] |
| **Gymnolaemata**  **Cheilostomata** | | | |
| Bugulidae | *Bugulina stolonifera* (Ryland, 1960) | https://doi.org/10.7910/DVN/SDJZ4X | Treibergs and Giribet [21] |
| Electridae | *Electra posidoniae* Gautier, 1954 | SAMN25648403 | Saadi et al. [20] |
| Membraniporidae | *Membranipora membranacea* (Linnaeus, 1767) | NCBI, SRA, SRR2131259 | Laumer et al. [22] |
| Schizoporellidae | *Schizoporella errata* (Waters, 1878) | NCBI, SRA, SRR11784297 | Santagata [23] |
| Watersiporidae | *Watersipora subtorquata* (d'Orbigny, 1852) | NCBI, SRA, SRR11783869 | Santagata [23] |
| **Gymnolaemata**  **Ctenostomata** | | |  |
| Hislopiidae | *Hislopia malayensis* Annandale, 1916 | NCBI, SRA, SAMN25648404 | Saadi et al. [20] |
| Monobryozoidae | *Monobryozoon ambulans* Remane, 1936 | NCBI, SRA, SRR25585376 | Current study |
| Paludicellidae | *Paludicella articulata* (Ehrenberg, 1831) | NCBI, SRA, SRR25585375 | Current study |
| Nolellidae | *Nolella* sp*.* | NCBI, SRA, SAMN25648405 | Saadi et al. [20] |
| Pherusellidae | *Pherusella minima* Decker, Gordon, Spencer Jones & Schwaha, 2021 | NCBI, SRA, SAMN25648406 | Saadi et al. [20] |
| Alcyonidiidae | *Alcyonidium polyoum* (Linnaeus, 1761) | NCBI, SRA, SAMN25648407 | Saadi et al. [20] |
|  | *Alcyonidium* sp. | NCBI, SRA, SRS14402042 | Drábková et al. [24] |
| Flustrellidridae | *Flustrellidra corniculata* (Smitt, 1872) | NCBI, SRA, SRR9667736 | Laumer et al. [18] |
|  | *Flustrellidra hispida* (Fabricius, 1780) | NCBI, SRA, SAMN25648408 | Saadi et al. [20] |
| **Outgroups:**  **Phoronida** | | | |
| Phoronidae | *Phoronis ovalis* Wright, 1856  AphiaID | NCBI, SRA, SAMN25648409 | Saadi et al. [20] |
|  | *Phoronis ijimai* Pixell, 1912 | NCBI, SRA, SRR1611566 | Halanych and Kocot [25] |
| **Brachiopoda** | | | |
| Laqueidae | *Laqueus erythraeus* (Koch, 1848) | NCBI, SRA, SRR1611557 | Halanych and Kocot [25] |
| Lingulidae | *Lingula anatina* Lamarck, 1801 | NCBI, TSA, GDJY01000001 | Luo et al. [26] |

**Table S2.** Tests of compositional homogeneity to determine whether models adequately represented the compositional variation of the data for both data matrices.

| **Complete data matrix** | | | | | | | | |
| --- | --- | --- | --- | --- | --- | --- | --- | --- |
| **CAT-F81** | | | | | **CAT-GTR** | | | |
| **Species** | **Observed (empirical) value)** | **Posterior predicted mean** | **z-score** | **P-value** | **Observed (empirical value)** | **Posterior predicted mean** | **z-score** | **P-value** |
| *Watersipora subtorquata* | 1.7631E-05 | 9.7692E-06 | 6.396 | 0 | 1.7631E-05 | 3.4287E-06 | 17.4315 | 0 |
| *Schizoporella errata* | 2.1824E-05 | 1.4014E-05 | 5.05368 | 0 | 2.1824E-05 | 4.4344E-06 | 16.6413 | 0 |
| *Electra posidoniae* | 5.5265E-05 | 6.6585E-06 | 46.3561 | 0 | 5.5265E-05 | 2.3294E-06 | 82.2472 | 0 |
| *Membranipora membranacea* | 5.1494E-05 | 8.5631E-06 | 36.3267 | 0 | 5.1494E-05 | 3.1079E-06 | 59.8325 | 0 |
| *Bugulina stolonifera* | 4.9476E-05 | 1.2244E-05 | 26.8623 | 0 | 4.9476E-05 | 3.8771E-06 | 50.0533 | 0 |
| *Hislopia malayensis* | 0.00010756 | 6.1306E-06 | 87.6676 | 0 | 0.00010756 | 2.7656E-06 | 122.362 | 0 |
| *Disporella hispida* | 3.2088E-05 | 3.5458E-06 | 36.8828 | 0 | 3.2088E-05 | 2.1655E-06 | 40.4398 | 0 |
| *Hirosella fruticosa* | 0.00031796 | 2.2195E-06 | 536.032 | 0 | 0.00031796 | 1.7075E-06 | 577.761 | 0 |
| *Fredericella sultana* | 0.00028447 | 2.7612E-06 | 378.689 | 0 | 0.00028447 | 1.9841E-06 | 419.844 | 0 |
| *Stephanella* cf. *hina* | 0.00018667 | 2.1901E-06 | 316.485 | 0 | 0.00018667 | 1.3133E-06 | 425.034 | 0 |
| *Phoronis ovalis* | 4.1814E-05 | 2.9319E-06 | 48.9899 | 0 | 4.1814E-05 | 2.3494E-06 | 54.9211 | 0 |
| *Phoronis Ijimai* | 7.3222E-05 | 3.5812E-06 | 76.3024 | 0 | 7.3222E-05 | 2.5212E-06 | 89.2386 | 0 |
| *Laqueus erythraeus* | 0.00015311 | 3.1125E-06 | 169.994 | 0 | 0.00015311 | 2.3666E-06 | 190.835 | 0 |
| *Lingula anatina* | 7.4913E-05 | 2.5062E-06 | 97.9137 | 0 | 7.4913E-05 | 1.8353E-06 | 111.002 | 0 |
| *Pherusella minima* | 0.00014279 | 1.687E-06 | 278.953 | 0 | 0.00014279 | 1.1924E-06 | 337.129 | 0 |
| *Flustrellidra hispida* | 0.00012707 | 1.8154E-06 | 233.412 | 0 | 0.00012707 | 1.2348E-06 | 288.842 | 0 |
| *Alcyonidium sp.* | 4.6031E-05 | 2.4486E-06 | 70.0235 | 0 | 4.6031E-05 | 1.448E-06 | 84.1003 | 0 |
| *Alcyonidium gelatinosum* | 3.2305E-05 | 1.7318E-06 | 60.2663 | 0 | 3.2305E-05 | 1.2775E-06 | 64.6301 | 0 |
| *Monobryozoon ambulan* | 3.0392E-05 | 5.7533E-06 | 19.8742 | 0 | 3.0392E-05 | 3.309E-06 | 26.7564 | 0 |
| *Nolella* sp*.* | 5.0474E-05 | 7.4063E-06 | 28.3756 | 0 | 5.0474E-05 | 4.492E-06 | 34.9706 | 0 |
| *Paludicella articulata* | 2.3208E-05 | 1.8123E-06 | 41.5842 | 0 | 2.3208E-05 | 1.0682E-06 | 52.284 | 0 |
| *Flustrellidra corniculata* | 0.00014507 | 1.8329E-06 | 289.616 | 0 | 0.00014507 | 1.3867E-06 | 288.5 | 0 |
| *Patinella* sp. | 0.00029575 | 0.00021811 | 5.45497 | 0 | 0.00029575 | 7.8047E-05 | 18.3546 | 0 |
| *Asajirella gelatinosa* | 0.00022879 | 1.4591E-06 | 484.106 | 0 | 0.00022879 | 1.1962E-06 | 570.76 | 0 |
| *Heteropora pacifica* | 0.00029238 | 0.00013198 | 12.8678 | 0 | 0.00029238 | 4.0439E-05 | 25.4223 | 0 |
| *Hyalinella punctata* | 0.00028548 | 5.1366E-06 | 267.017 | 0 | 0.00028548 | 3.3558E-06 | 289.176 | 0 |
| *Plumatella javanica* | 0.0002752 | 2.8423E-06 | 349.727 | 0 | 0.0002752 | 2.4957E-06 | 345.477 | 0 |
| **Subsampled data matrix** | | | | | | | | |
| CAT-F81 | | | | | CAT-GTR | | | |
| *Watersipora subtorquata* | 0.00011682 | 1.6167E-06 | 202.886 | 0 | 0.00011682 | 1.6167E-06 | 202.886 | 0 |
| *Schizoporella errata* | 8.3029E-05 | 1.2764E-06 | 168.231 | 0 | 8.3029E-05 | 1.2764E-06 | 168.231 | 0 |
| *Electra posidoniae* | 0.00013661 | 1.4937E-06 | 234.695 | 0 | 0.00013661 | 1.4937E-06 | 234.695 | 0 |
| *Membranipora membranacea* | 3.8559E-05 | 3.2573E-06 | 50.9624 | 0 | 3.8559E-05 | 3.2573E-06 | 50.9624 | 0 |
| *Bugulina stolonifera* | 1.7989E-05 | 1.2363E-06 | 40.9148 | 0 | 1.7989E-05 | 1.2363E-06 | 40.9148 | 0 |
| *Hislopia malayensis* | 1.8775E-05 | 2.6769E-06 | 17.7835 | 0 | 1.8775E-05 | 2.6769E-06 | 17.7835 | 0 |
| *Disporella hispida* | 2.1405E-05 | 1.624E-06 | 37.6758 | 0 | 2.1405E-05 | 1.624E-06 | 37.6758 | 0 |
| *Hirosella fruticosa* | 5.4476E-05 | 2.1003E-06 | 68.6007 | 0 | 5.4476E-05 | 2.1003E-06 | 68.6007 | 0 |
| *Fredericella sultana* | 0.00018303 | 1.796E-06 | 295.817 | 0 | 0.00018303 | 1.796E-06 | 295.817 | 0 |
| *Stephanella* cf. *hina* | 2.0814E-05 | 5.9497E-06 | 11.9624 | 0 | 2.0814E-05 | 5.9497E-06 | 11.9624 | 0 |
| *Phoronis ovalis* | 1.3912E-05 | 4.637E-06 | 9.94772 | 0 | 1.3912E-05 | 4.637E-06 | 9.94772 | 0 |
| *Phoronis Ijimai* | 0.00024172 | 3.5691E-05 | 25.2707 | 0 | 0.00024172 | 3.5691E-05 | 25.2707 | 0 |
| *Laqueus erythraeus* | 0.00028833 | 8.7683E-05 | 16.0119 | 0 | 0.00028833 | 8.7683E-05 | 16.0119 | 0 |
| *Lingula anatina* | 0.00015981 | 2.4753E-06 | 178.507 | 0 | 0.00015981 | 2.4753E-06 | 178.507 | 0 |
| *Pherusella minima* | 7.1587E-05 | 2.3493E-06 | 94.3633 | 0 | 7.1587E-05 | 2.3493E-06 | 94.3633 | 0 |
| *Flustrellidra hispida* | 5.0646E-05 | 2.7988E-06 | 49.1381 | 0 | 5.0646E-05 | 2.7988E-06 | 49.1381 | 0 |
| *Alcyonidium* sp. | 0.00016535 | 3.0798E-06 | 168.637 | 0 | 0.00016535 | 3.0798E-06 | 168.637 | 0 |
| *Alcyonidium gelatinosum* | 2.5187E-05 | 2.401E-06 | 30.0901 | 0 | 2.5187E-05 | 2.401E-06 | 30.0901 | 0 |
| *Monobryozoon ambulans* | 3.3565E-05 | 3.8353E-06 | 30.1252 | 0 | 3.3565E-05 | 3.8353E-06 | 30.1252 | 0 |
| *Nolella* sp*.* | 8.6768E-05 | 1.8965E-06 | 129.804 | 0 | 8.6768E-05 | 1.8965E-06 | 129.804 | 0 |
| *Paludicella articulata* | 3.5985E-05 | 4.3622E-06 | 29.1832 | 0 | 3.5985E-05 | 4.3622E-06 | 29.1832 | 0 |
| *Flustrellidra corniculata* | 0.00015934 | 4.0357E-06 | 132.63 | 0 | 0.00015934 | 4.0357E-06 | 132.63 | 0 |
| *Patinella* sp. | 2.8636E-05 | 3.2739E-06 | 20.0626 | 0 | 2.8636E-05 | 3.2739E-06 | 20.0626 | 0 |
| *Asajirella gelatinosa* | 0.00011313 | 3.0646E-06 | 122.913 | 0 | 0.00011313 | 3.0646E-06 | 122.913 | 0 |
| *Heteropora pacifica* | 6.9902E-05 | 1.3565E-06 | 132.601 | 0 | 6.9902E-05 | 1.3565E-06 | 132.601 | 0 |
| *Hyalinella punctata* | 3.1393E-05 | 1.8467E-06 | 50.648 | 0 | 3.1393E-05 | 1.8467E-06 | 50.648 | 0 |
| *Plumatella javanica* | 3.1983E-05 | 2.9207E-06 | 31.4251 | 0 | 3.1983E-05 | 2.9207E-06 | 31.4251 | 0 |

**Table S3**: Detailed results of PPA analyses related to Table 1. This table illustrates the empirical heterogeneity observed directly from the data, the average posterior predictive mean and the dispersion around the mean for the mean amino for the five PPA statistics of the complete dataset.

**Table S4**: Detailed results of PPA analyses related to Table 1. This table illustrates the empirical heterogeneity observed directly from the data, the average posterior predictive mean and the dispersion around the mean for the mean amino for the five PPA statistics of the subsampled dataset.

**Table S5.** Fossil calibration nodes used. Node numbers correspond to nodes labels in Figure 7. The second column gives the input used in MCMCTree for the Cauchy “L”, skew normal “SN” and uniform “B” prior age distributions.

| Node | MCMCTree input | Placement on the input tree |
| --- | --- | --- |
| 1 | L(5.09, 0.025, 0.061, 1e-300)  B(5.09, 6.36, 0.025, 0.025)  SN(5.14,0.54,50) | The calibrated node is placed at the split between *Lingula anatine* and *Laqueus erythraeus* |
| 2 | L(4.78, 0.025, 0.074, 1e-300)  B(4.78, 6.36, 0.025, 0.025)  SN(4.83,0.68,50) | The calibrated node is placed at the split between cyclostomes and cheilostomes+ ctenostomes |
| 3 | L(2.37, 0.025, 0.17, 1e-300)  B(2.37, 6.36, 0.025, 0.025)  SN(2.42,1.76,50) | The calibrated node is placed at the stem cyclostomes |
| 4 | L(1. 36, 0.025, 0.21, 1e-300)  B(1.36, 6.36, 0.025, 0.025)  SN(1.41,2,50) | The calibrated node is placed at the stem of phylactolaemate |
| 5 | L(1.50, 0.025, 0.205, 1e-300)  B(1.50, 6.36, 0.025, 0.025)  SN(1.55,2,50) | The calibrated node is placed at the stem of cheilostomes |

**Table S6**. Summary statistics of the transcriptome assembly for *Monobryozoon ambulans*.

| **Transcriptome feature** | **Values** |
| --- | --- |
| Number of reads before processing | 23,831,121 |
| Number of reads after processing | 19,782,297 |
| Assembled contigs | 102,642 |
| Number of transcripts > 1,000 bp | 23,079 |
| Number of reconstructed bases (bp) | 69,524,197 |
| Number of reconstructed bases in transcripts reconstructed >1,000 bp | 35,240,931 |
| N50 | 1,197 |
| GC content | 41.28% |
| Number of possible putative proteins | 50,309 |
| Number of selected putative proteins | 36,428 |
| Number of non-redundant putative proteins | 22,693 |
| BUSCOs completeness (%) | 74.8% |
| Complete BUSCOs | 713 |
| Complete single-copy BUSCOs | 635 (66.6%) |
| Complete and duplicated BUSCOs | 78 (8.2%) |
| Fragmented BUSCOs | 130 (13.2%) |
| Missing BUSCOs | 111 (12.8%) |

**Table S7**. Number of unique and total functional annotation of the *Monobryozoon ambulans* transcriptome using Trinotate pipeline.

| **Annotation Results** | **Unique Number of Sequences** | **Total Number of Sequences** |
| --- | --- | --- |
| Protein hits (BLASTx-Uniref90) | 42,863 | 44,069 |
| Protein hits (BLASTx-Uniprot/Swissprot) sprot_Top_BLASTX_hit | 39,842 | 41,092 |
| gene_ontology_blast | 11,315 | 32,029 |
| EggNOG | 4,172 | 26,680 |
| KEGG | 11,893 | 28,408 |
| Protein hits (BLASTP-Uniref90) | 26,172 | 32,129 |
| Protein hits (BLASTP-Uniprot/Swissprot) sprot_Top_BLASTP_hit | 18,880 | 23,428 |
| Pfam | 21,977 | 44,078 |
| GO Pfam gene_ontology_Pfam | 14,79 | 16,904 |
| TmHMM | 5,112 | 6,434 |
| SignalP | 10,940 | 14,771 |
| RNAMMER | 60 | 65 |

**
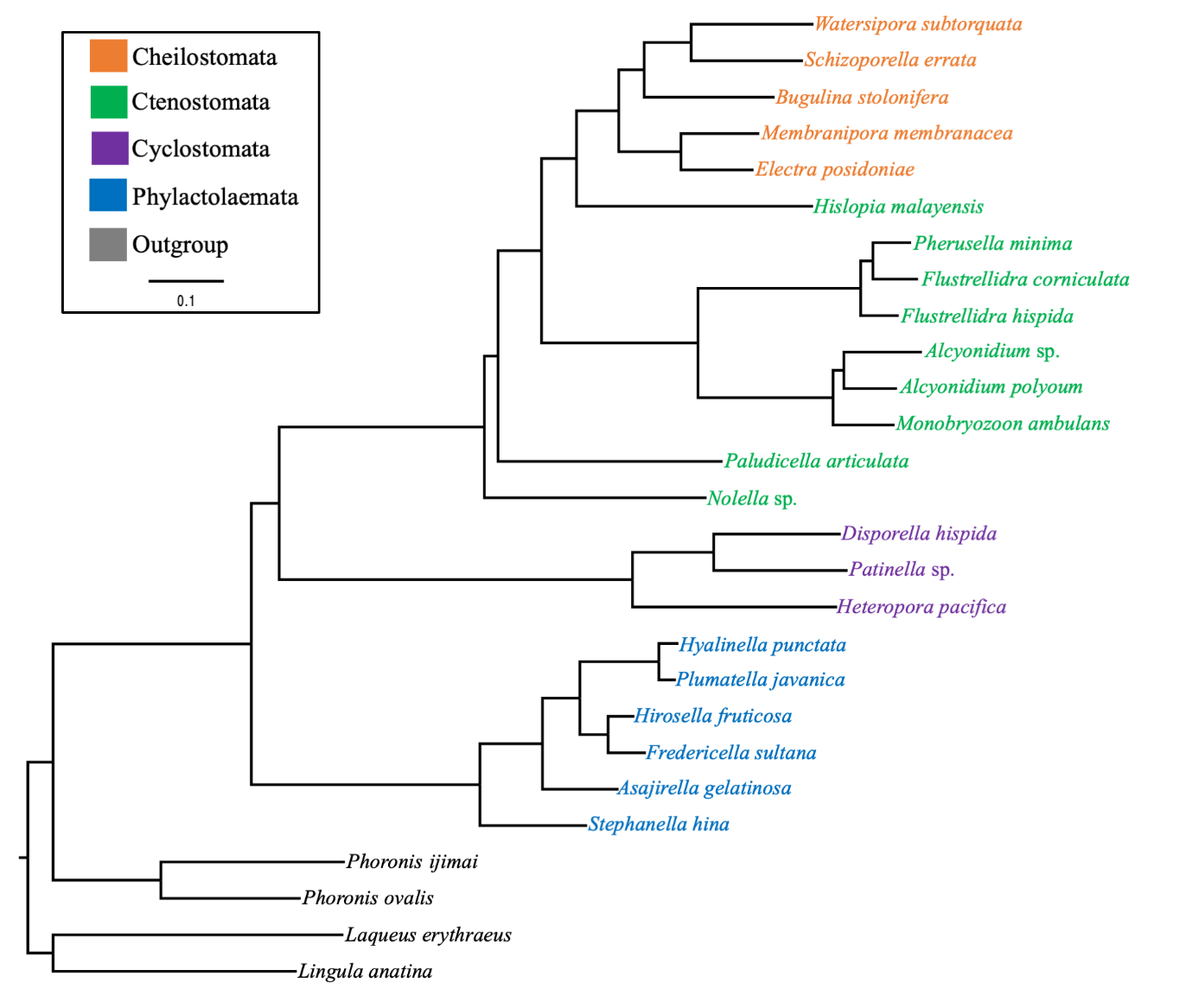
**

**Figure S1:** Maximum likelihood phylogeny of Bryozoa based on the complete data matrix, including 422,961 AAs from 2,014 OGs using unpartitioned analysis with PMSF model. All nodes are supported by 100 ultrafast bootstraps. The scale bar represents 1 substitutional change per 100 AAs.


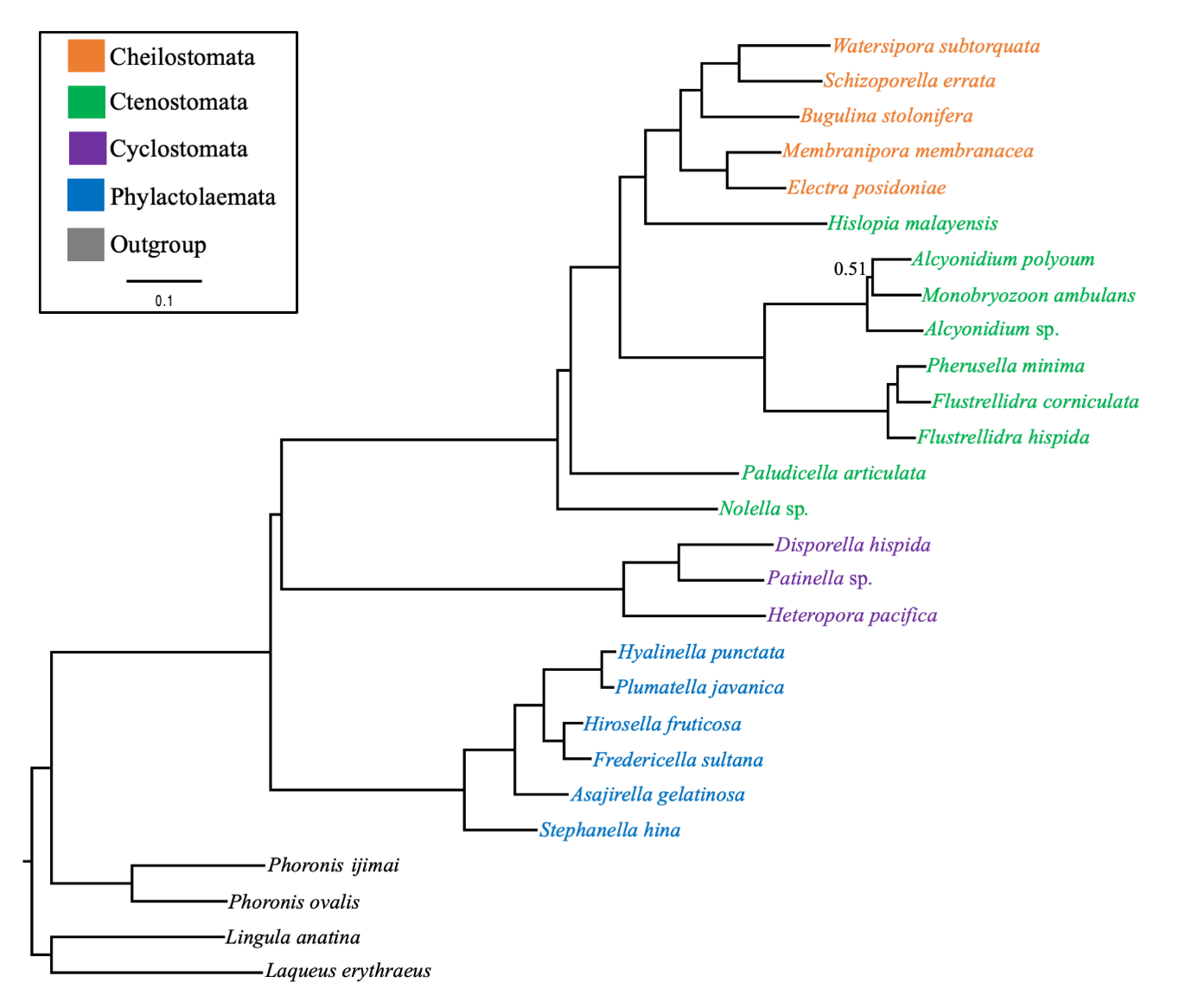


**Figure S2:** Bayesian inference tree of Bryozoa based on the complete data matrix, including 422,961 AAs from 2,014 OGs with CAT-F81+G model. Bayesian posterior probabilities are only shown for nodes that are not maximally supported. The scale bar represents 1 substitutional change per 100 AAs.


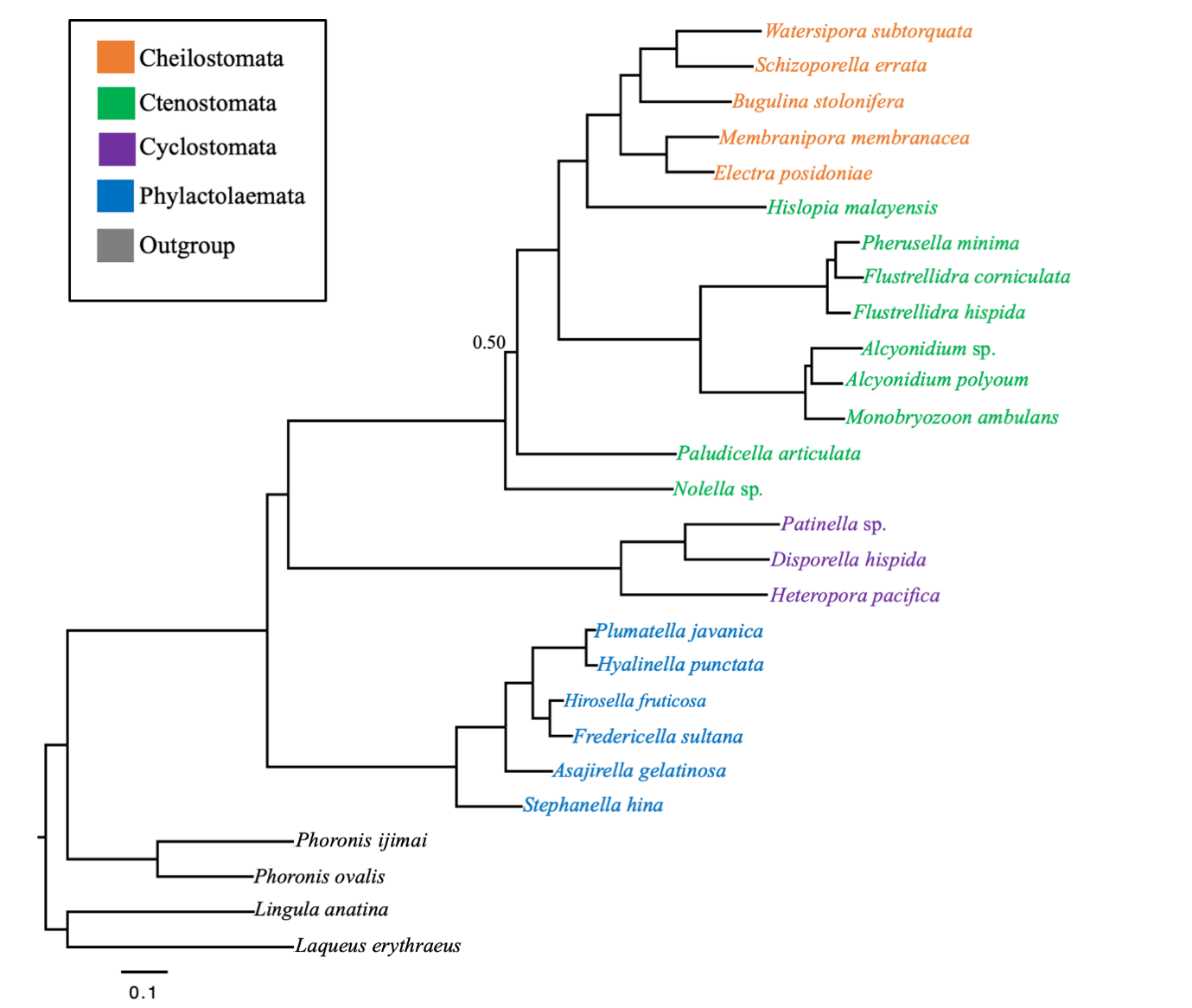


**Figure S3:** Bayesian inference tree of Bryozoa based on the complete data matrix, including 422,961 AAs from 2,014 OGs with CAT-GTR+G model. Bayesian posterior probabilities are only shown for nodes that are not maximally supported. The scale bar represents 1 substitutional change per 100 AAs.


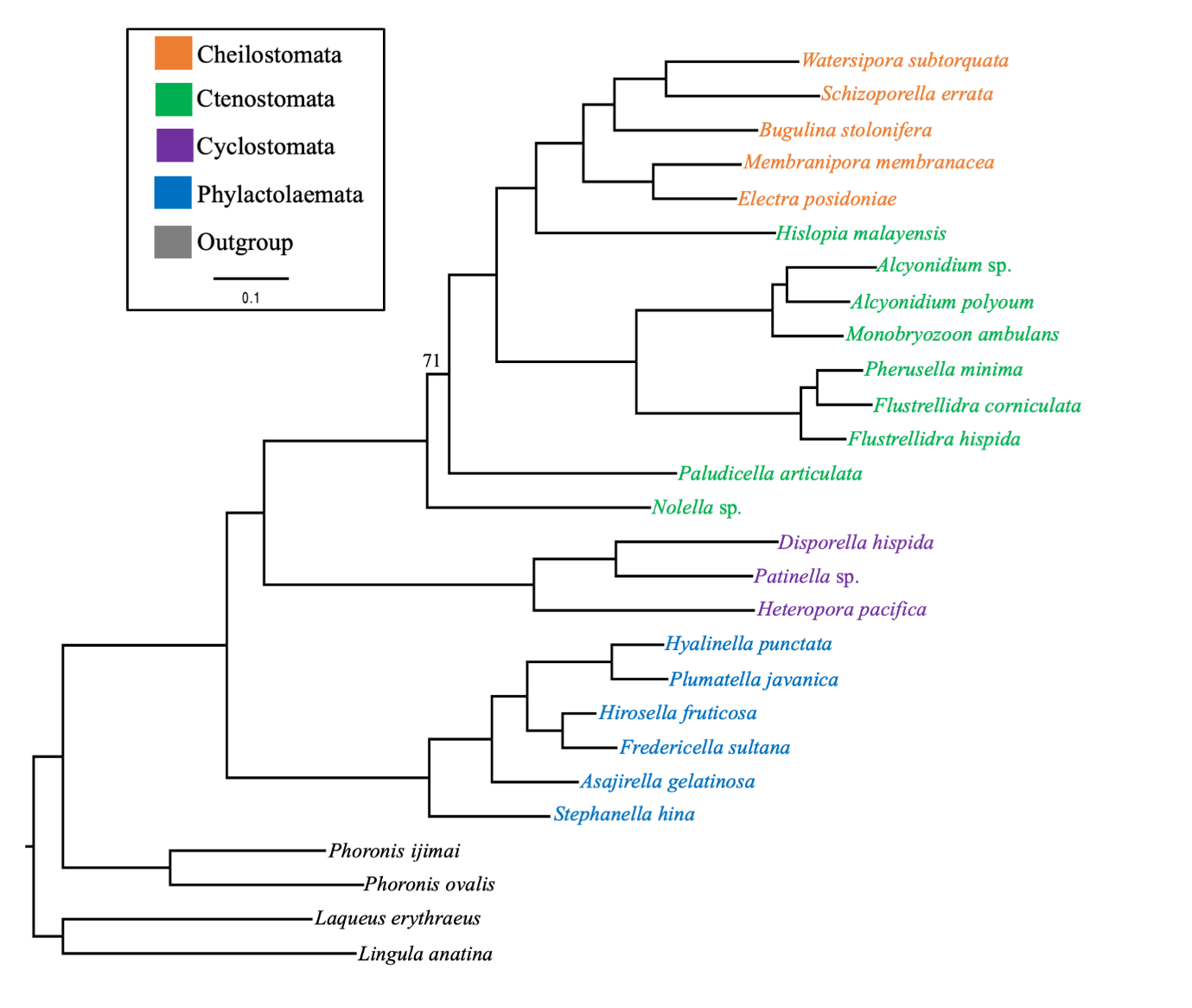


**Figure S4:** Maximum likelihood phylogeny of Bryozoa based on the subsampled data matrix, including 310,190 AAs from 1500 OGs using partitioned analysis. Values on some nodes represent ML ultrafast bootstrap support and only shown for nodes that are not maximally supported by all analyses. The scale bar represents 1 substitutional change per 100 AAs.


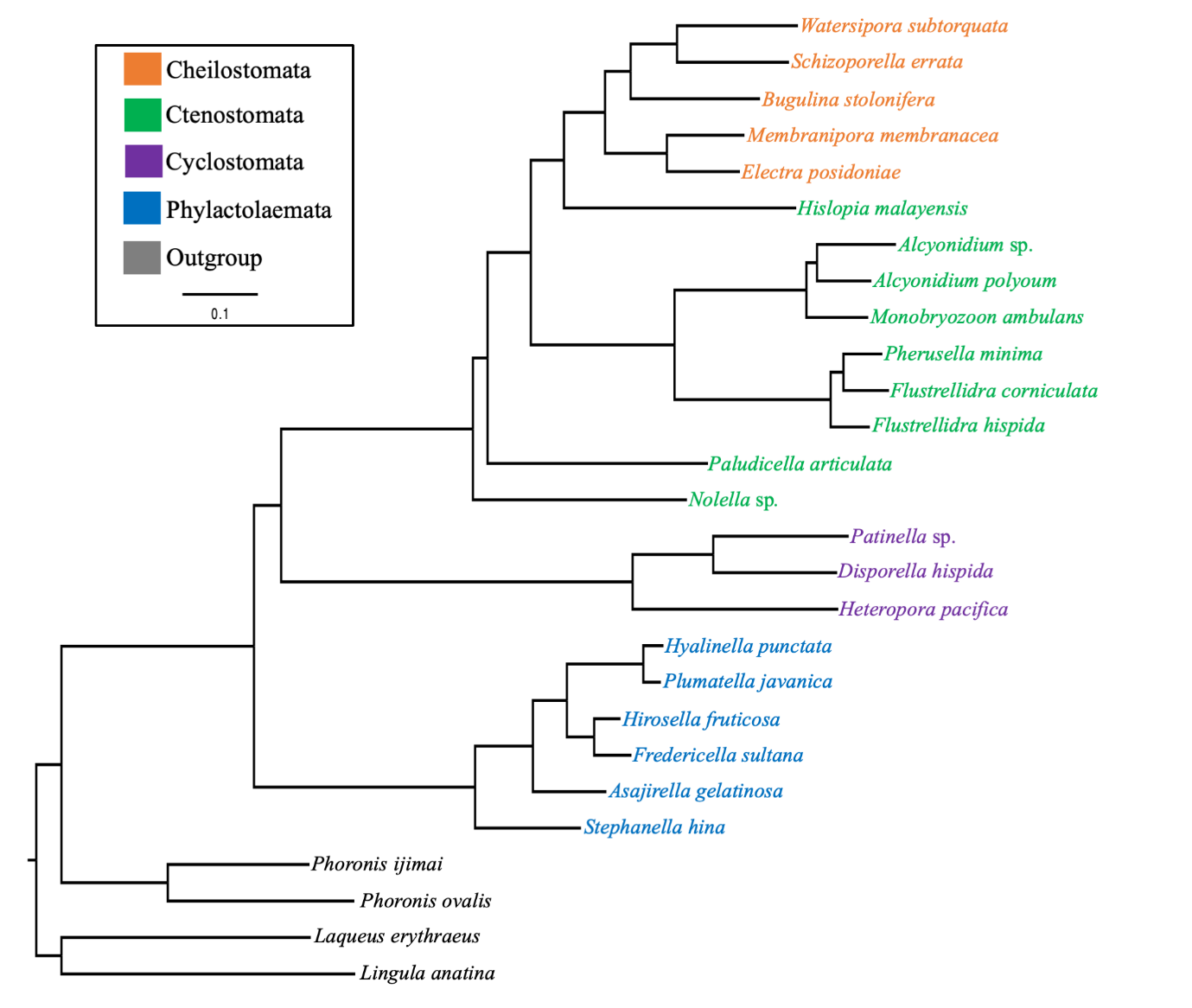


**Figure S5:** Maximum likelihood phylogeny of Bryozoa based on the subsampled data matrix, including 310,190 AAs from 1500 OGs using unpartitioned analysis with PMSF model. All nodes are supported by 100 ultrafast bootstraps. The scale bar represents 1 substitutional change per 100 AAs.


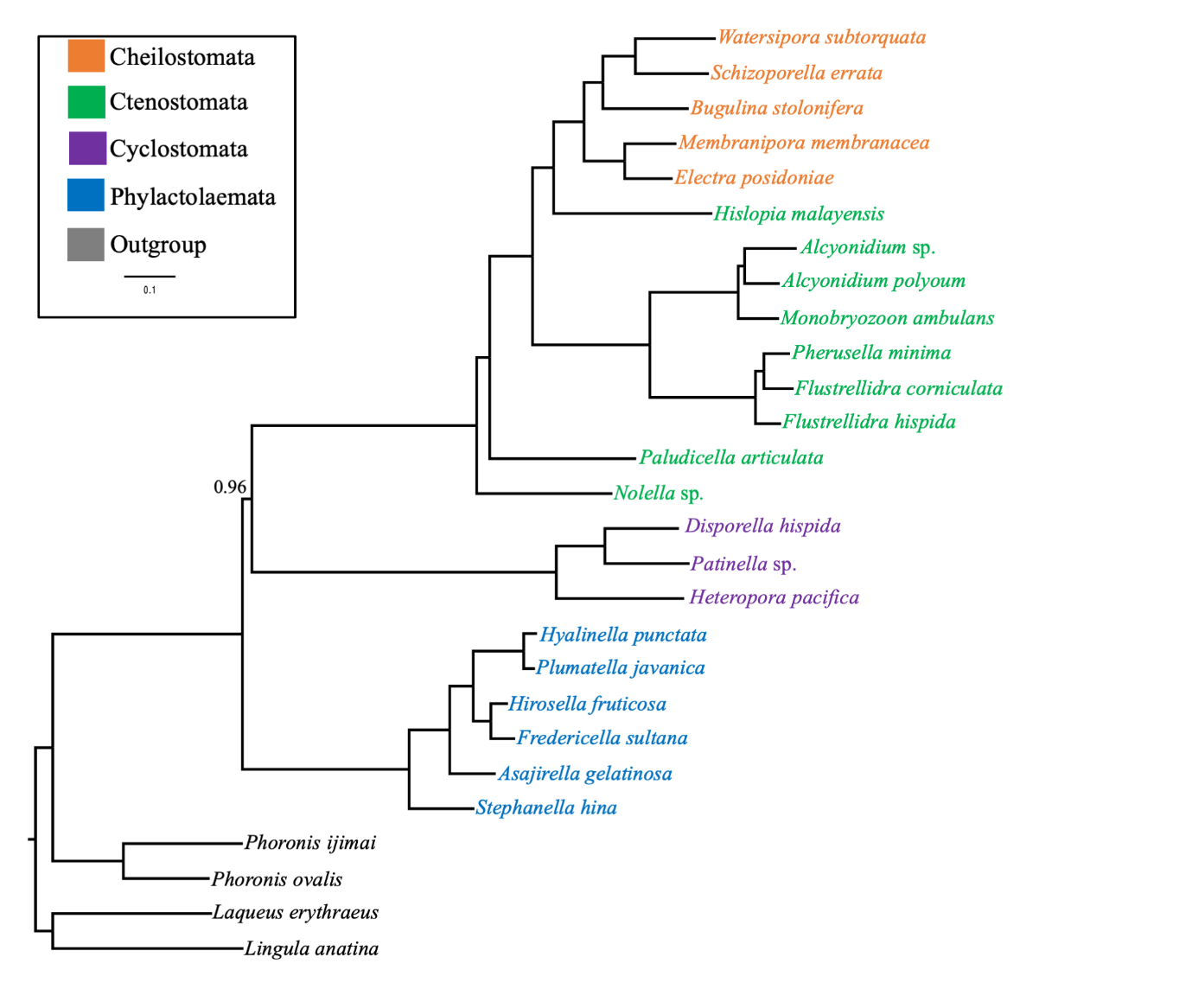


**Figure S6:** Bayesian inference tree of Bryozoa based on the subsampled data matrix, including 310,190 AAs from 1500 OGs with CAT-F81+G model. Bayesian posterior probabilities are only shown for nodes that are not maximally supported. The scale bar represents 1 substitutional change per 100 AAs.


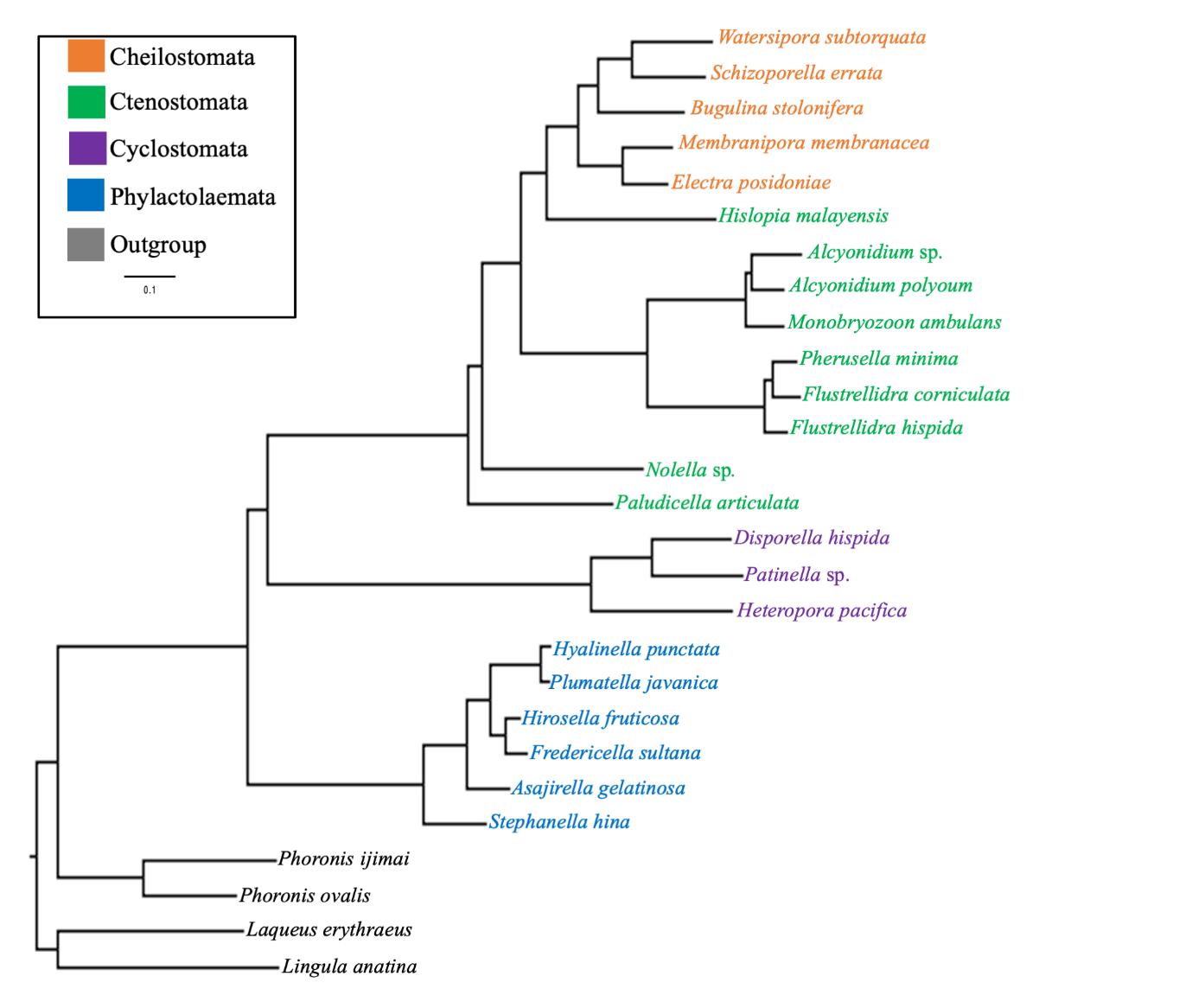


**Figure S7:** Bayesian inference tree of Bryozoa based on the subsampled data matrix, including 310,190 AAs from 1500 OGs with CAT-GTR+G model. Bayesian posterior probabilities are only shown for nodes that are not maximally supported. The scale bar represents 1 substitutional change per 100 AAs.


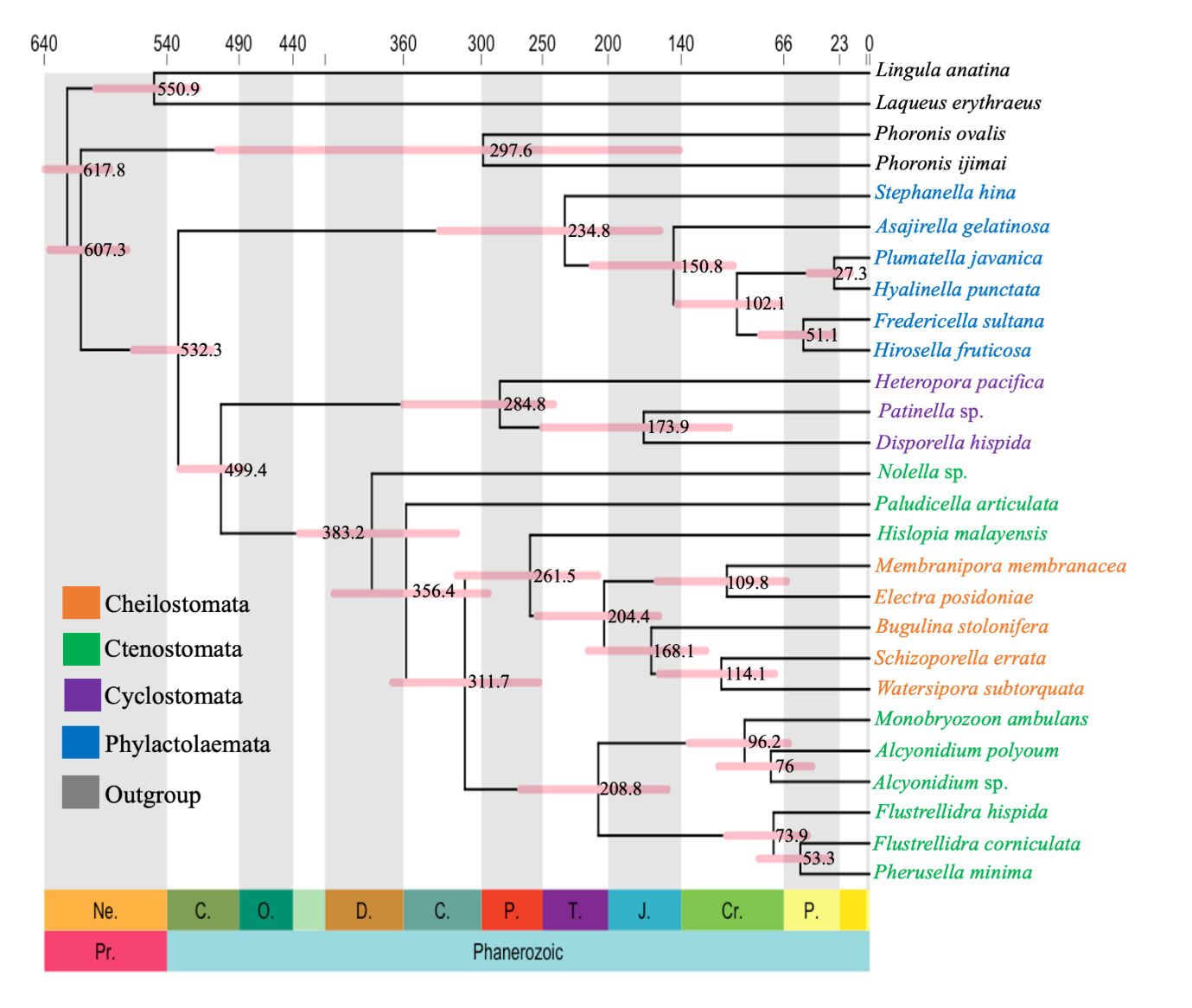


**Figure S8.** Time-calibrated phylogeny of Bryozoa using MCMCTree based on the complete data matrix with posterior distributions based on the skew normal priors. A time scale in Ma is shown above the tree, with geographical periods labelled below the tree. Node bars represent 95% confidence intervals of age estimates and raw numbers for mean.


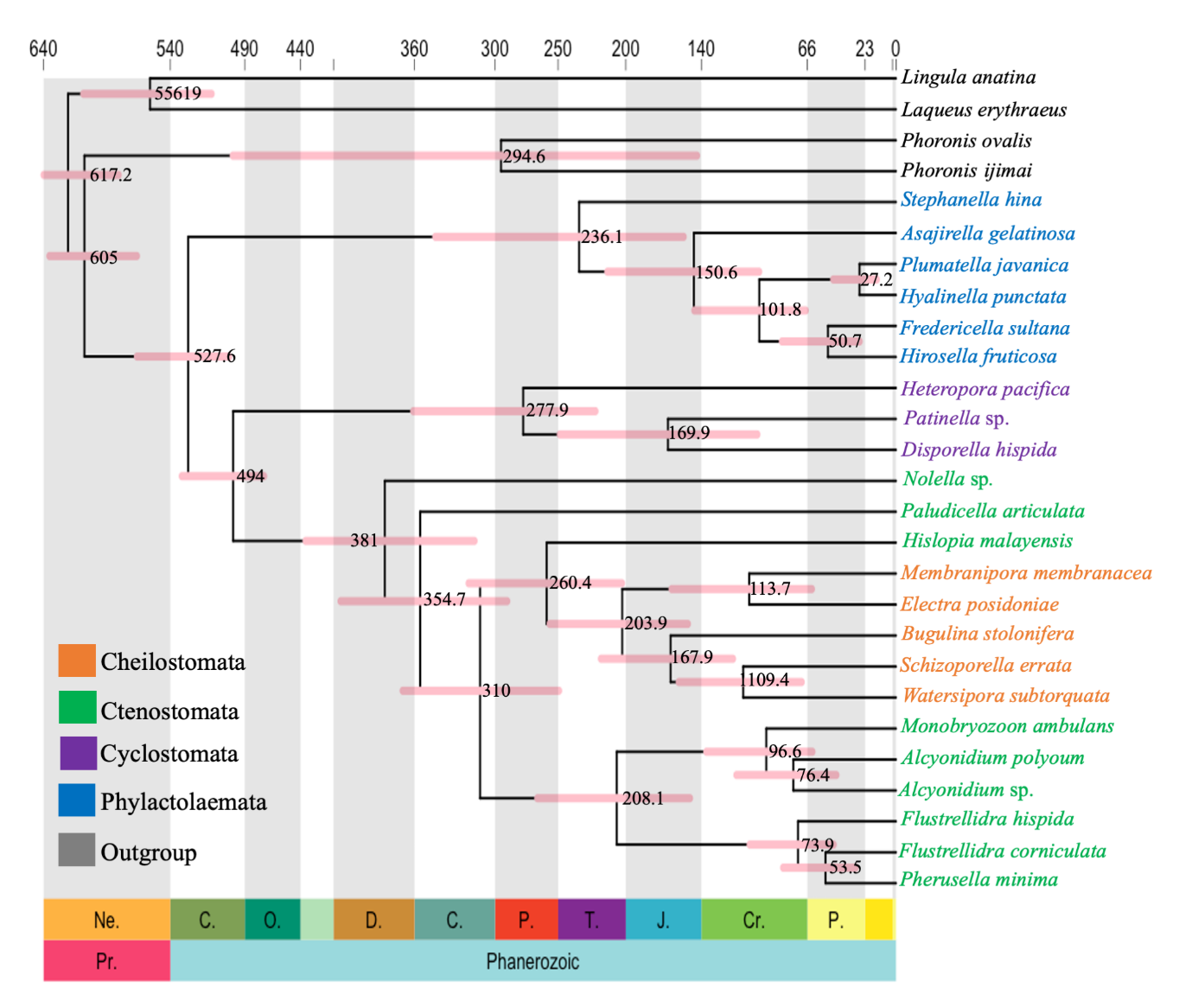


**Figure S9**. Time-calibrated phylogeny of Bryozoa using MCMCTree based on the complete data matrix with posterior distributions based on the uniform priors. A time scale in Ma is shown above the tree, with geographical periods labelled below the tree. Node bars represent 95% confidence intervals of age estimates and raw numbers for mean.


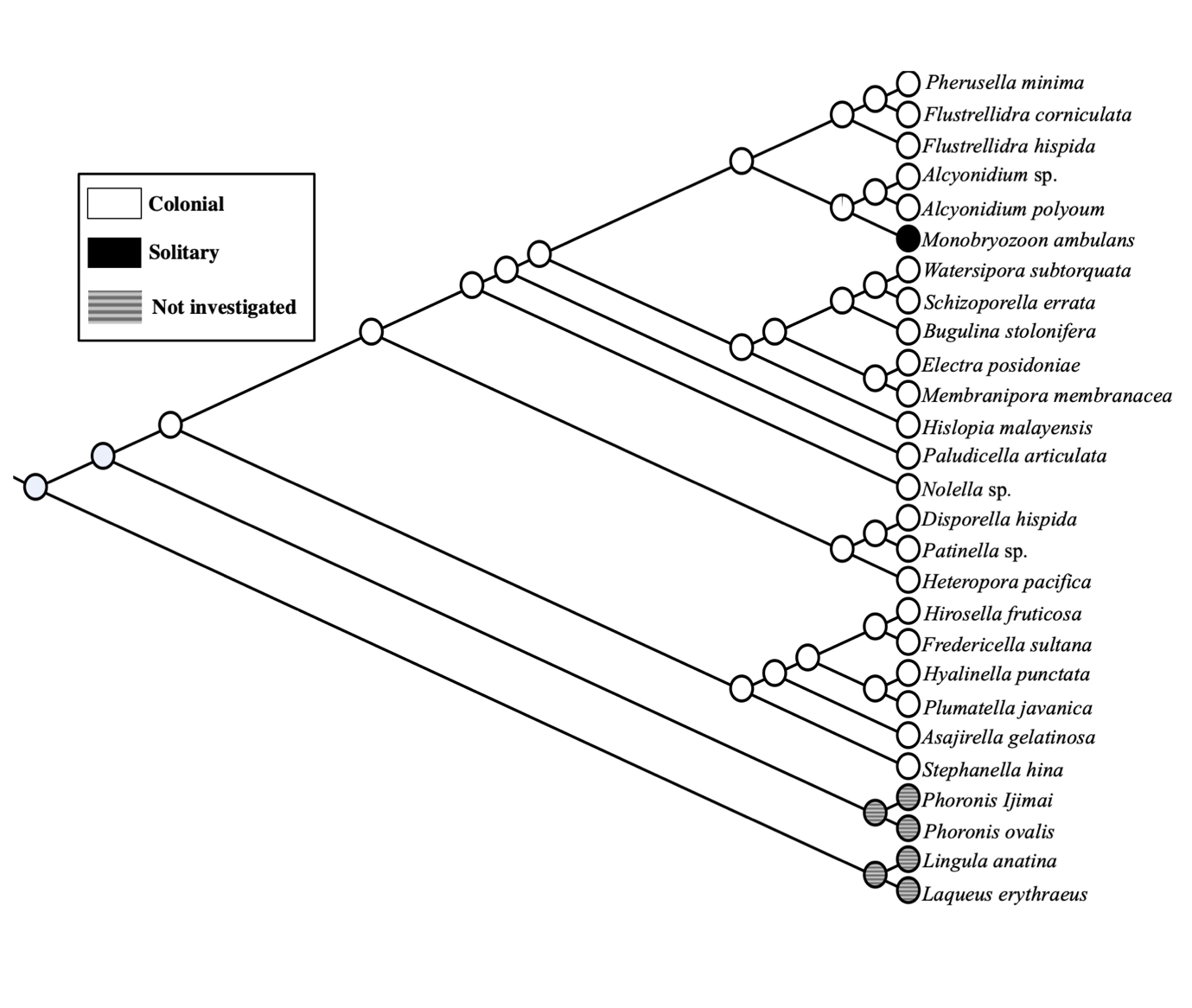


**Figure S10.** Ancestral state reconstruction for the lifestyles in bryozoans. The pie area indicates the likelihood of character state at each node.


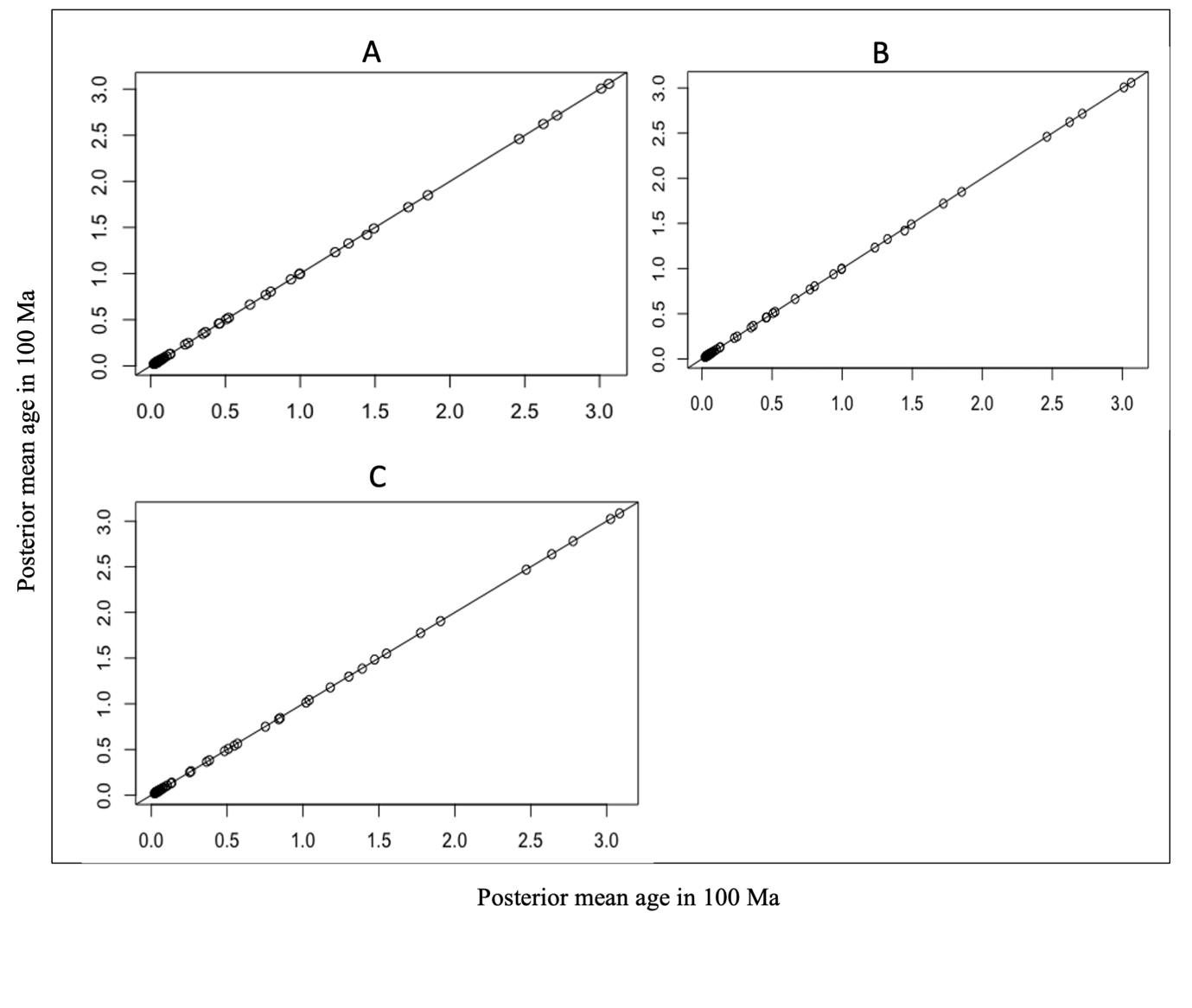


**Figure S11.** Convergence plots for the divergence time analyses showing the relationship between the posterior mean of the two runs of each calibration strategy (A Cauchy, B skew normal and C uniform).


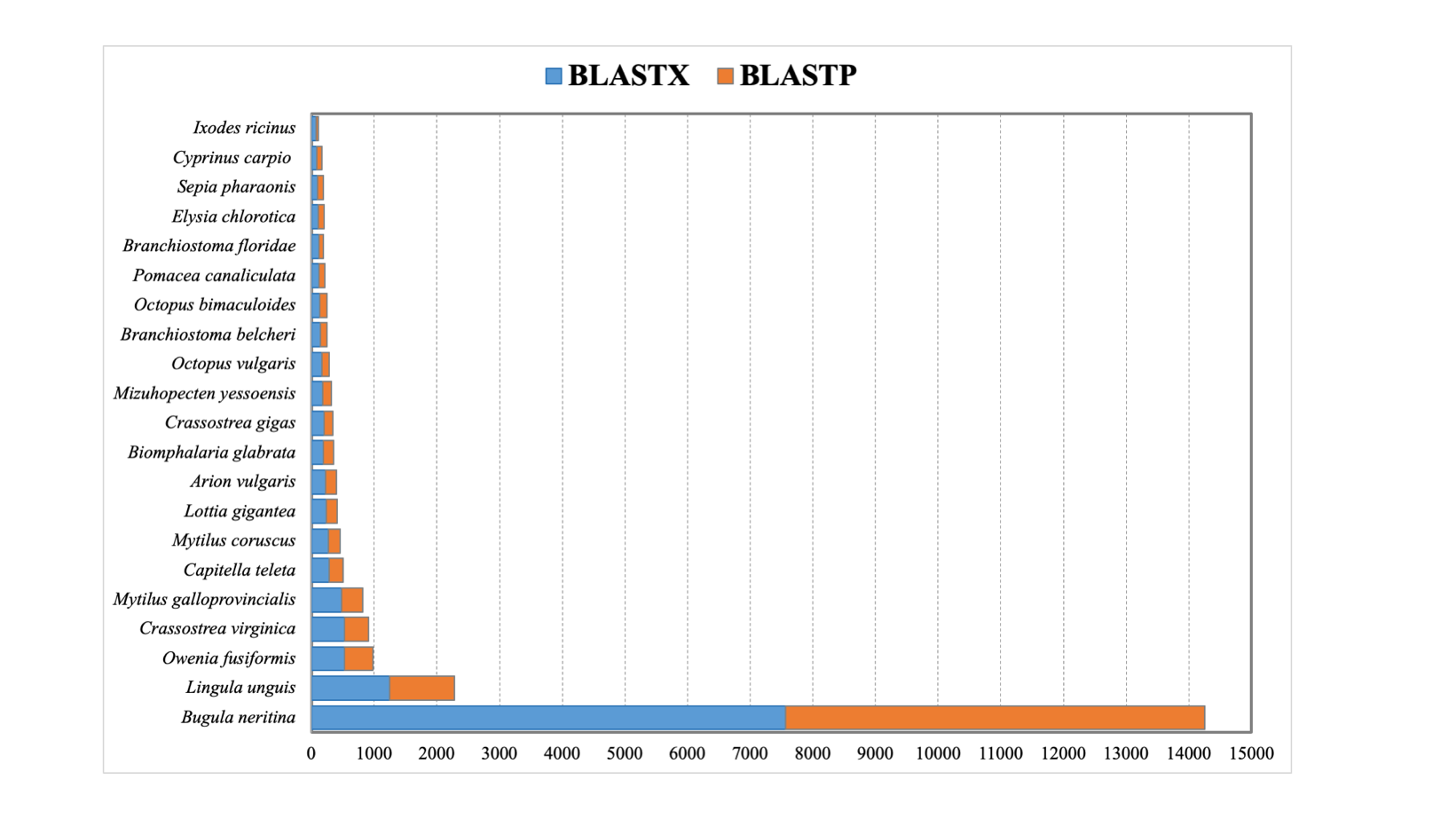


**Figure S12**. Top 20 species taxonomic distribution on the basis of BLASTX and BLASTP hits of *Monobryozoon ambulans* transcriptome against UniRef90 database.


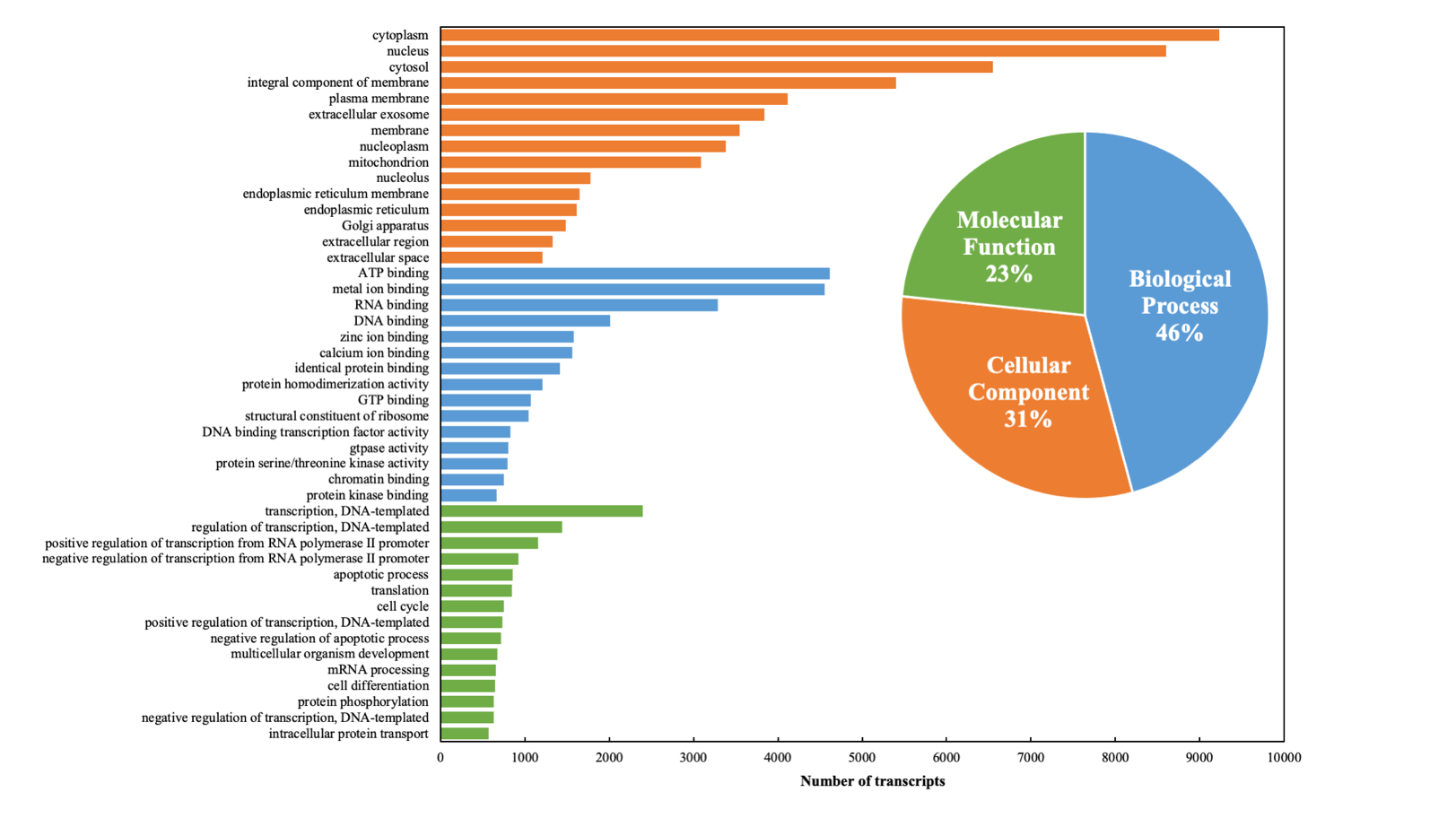


**Figure S13**: GO functional annotation of *Monobryozoon ambulans* transcriptome, where orange represents cellular component, blue represents biological process, and green represents molecular function. The Y-axis represents distribution of the top 15 GO terms of each category, the X- the number of transcripts. Pie-charts showing the percentage of three GO categories.

**Figure S14**: Classification of eggNOG annotations of the *Monobryozoon ambulans* transcriptome. The capital letters on the Y-axis represent different eggNOG categories. X-axis shows the number of transcripts in each eggNOG category.

**References**

1. Altschul SF, Gish W, Miller W, Myers EW, Lipman DJ. Basic local alignment search tool. J Mol Biol. 1990; 215: 403-410. doi: 10.1016/s0022-2836(05)80360-2.

2. Lagesen K, Hallin P, Rødland EA, Staerfeldt HH, Rognes T, Ussery DW. RNAmmer: consistent and rapid annotation of ribosomal RNA genes. Nucleic Acids Res. 2007; 35: 3100-3108. doi: 10.1093/nar/gkm160.

3. Finn RD, Clements J, Eddy SR. HMMER web server: interactive sequence similarity searching. Nucleic Acids Res. 2011; 39: W29-37.

4. Petersen TN, Brunak S, von Heijne G, Nielsen H. SignalP 4.0: discriminating signal peptides from transmembrane regions. Nat Methods 2011; 8: 785-786. doi: 10.1038/nmeth.1701.

5. Krogh A, Larsson B, von Heijne G, Sonnhammer EL Predicting transmembrane protein topology with a hidden Markov model: application to complete genomes. J Mol Biol. 2001; 305: 567-580. doi: 10.1006/jmbi.2000.4315.

6. Ushatinskaya G. Origin and dispersal of the earliest brachiopods. Paleontological Journal 2008; 42: 776-791.

7. Kouchinsky A, Bengtson S, Landing E, Steiner M, Vendrasco M, Ziegler K. Terreneuvian stratigraphy and faunas from the Anabar Uplift, Siberia. Acta Palaeontologica Polonica. 2017; 62.

8. Benton MJ, Donoghue PCJ, Asher RJ. Calibrating and constraining molecular clocks. In Hedges KS, Blair S. (eds) The Timetree of Life. Oxford, UK: Oxford University Press. 2009; 35–86.

9. Pettersson Stolk S, Holmer LE, Caron JB. First record of the brachiopod *Lingulella waptaensis* with pedicle from the Middle Cambrian Burgess Shale. Acta Zoologica. 2010; 91: 150-162.

10. Dzik, J. (1981). Evolutionary relationships of the Early Paleozoic cyclostomatous Bryozoa. Palaeontology, 24, 827-862.

11. Taylor PD, Michalik K. Cyclostome bryozoans from the Late Triassic (Rhaetian) of the West Carpathians, Czechoslovakia. Neues Jahrbuch für Geologie und Paläontologie Abhandlungen 1991; 182: 285-302.

12. Vinogradov AV. New fossil freshwater bryozoans from the Asiatic part of Russia and Kazakhstan. Paleontological Journal 1996; 30: 284-292.

13. Pohowsky RA. A Jurassic cheilostome from England. In Larwood GP (ed). Living and fossil Bryozoa.London: Academic Press. 1973; 447-461.

14. Kalyaanamoorthy S, Minh BQ, Wong TKF, von Haeseler A, Jermiin LS. ModelFinder: fast model selection for accurate phylogenetic estimates. Nat Methods 2017; 14: 587-589. doi: 10.1038/nmeth.4285.

15. Nascimento FF, Reis MD, Yang Z. A biologist's guide to Bayesian phylogenetic analysis. Nat Ecol Evol. 2017; 1: 1446-1454. doi: 10.1038/s41559-017-0280-x.

16. Puttick MN. MCMCtreeR: functions to prepare MCMCtree analyses and visualize posterior ages on trees. Bioinformatics 2019; 35: 5321-5322. doi: 10.1093/bioinformatics/btz554.

17. Simão FA, Waterhouse RM, Ioannidis P, Kriventseva EV, Zdobnov EM. BUSCO: assessing genome assembly and annotation completeness with single-copy orthologs. Bioinformatics 2015; 31: 3210-3212. doi: 10.1093/bioinformatics/btv351.

18. Laumer CE, Fernández R, Lemer S, Combosch D, Kocot KM, Riesgo A, Andrade SCS, Sterrer W, Sørensen MV, Giribet G. Revisiting metazoan phylogeny with genomic sampling of all phyla. Proc Biol Sci. 2019; 286: 20190831. doi: 10.1098/rspb.2019.0831.

19. Kumar G, Ertl R, Bartholomew JL, El-Matbouli M. First transcriptome analysis of bryozoan *Fredericella sultana*, the primary host of myxozoan parasite *Tetracapsuloides bryosalmonae*. PeerJ 2020 8**,** e9027-e9027. doi: 10.7717/peerj.9027.

20. Saadi AJ, Bibermair J, Kocot KM, Roberts NG, Hirose M, Calcino A, Baranyi C, Chaichana R, Wood TS, Schwaha T. Phylogenomics reveals deep relationships and diversification within phylactolaemate bryozoans. Proc Biol Sci 2022; 289: 20221504. doi: 10.1098/rspb.2022.1504.

21. Treibergs KA, Giribet G. Differential gene expression between polymorphic zooids of the marine bryozoan *Bugulina stolonifera*. G3 (Bethesda) 2020; 10: 3843-3857. doi: 10.1534/g3.120.401348.

22. Laumer CE, Bekkouche N, Kerbl A, Goetz F, Neves RC, Sørensen MV, Kristensen RM, Hejnol A, Dunn CW, Giribet G, Worsaae K. Spiralian phylogeny informs the evolution of microscopic lineages. Curr Biol 2015; 25: 2000-2006. doi: 10.1016/j.cub.2015.06.068.

23. Santagata S. Genes with evidence of positive selection as potentially related to coloniality and the evolution of morphological features among the lophophorates and entoprocts. J Exp Zool B Mol Dev Evol 2021; 336: 267-280. doi: 10.1002/jez.b.22975.

24. Drábková M, Kocot KM, Halanych KM, Oakley TH, Moroz LL, Cannon JT, Kuris A, Garcia-Vedrenne AE, Pankey MS, Eliis EA, Varney R, Stefka J, Zrzavy J. Different phylogenomic methods support monophyly of enigmatic 'Mesozoa' (Dicyemida + Orthonectida, Lophotrochozoa). Proc Biol Sci 2022; 289: 20220683. doi: 10.1098/rspb.2022.0683.

25. Halanych KM, Kocot KM. Repurposed transcriptomic data facilitate discovery of innate immunity toll-like receptor (TLR) Genes across Lophotrochozoa. Biol Bull 2014; 227: 201-209. doi: 10.1086/BBLv227n2p201.

26. Luo YJ, Takeuchi T, Koyanagi R, Yamada L, Kanda M, Khalturina M, Fujie M, Yamasaki F, Endo K, Satoh N. The *Lingula* genome provides insights into brachiopod evolution and the origin of phosphate biomineralization. Nat Commun 2015; 6**:** 8301. doi: 10.1038/ncomms9301.
